# Analysis and correction of errors in nanoscale particle tracking using the Single-pixel interior filling function (SPIFF) algorithm

**DOI:** 10.1038/s41598-017-14166-6

**Published:** 2017-11-29

**Authors:** Yuval Yifat, Nishant Sule, Yihan Lin, Norbert F. Scherer

**Affiliations:** 1 James Franck Institute, The University of Chicago, Chicago Il, 60637 USA; 2 Department of Chemistry, The University of Chicago, Chicago Il, 60637 USA; 3 Center for Quantitative Biology, Peking-Tsinghua Center for Life Sciences, Academy for Advanced Interdisciplinary Studies, Peking University, Beijing, 100871 China

## Abstract

Particle tracking, which is an essential tool in many fields of scientific research, uses algorithms that retrieve the centroid of tracked particles with sub-pixel accuracy. However, images in which the particles occupy a small number of pixels on the detector, are in close proximity to other particles or suffer from background noise, show a systematic error in which the particle sub-pixel positions are biased towards the center of the pixel. This “pixel locking” effect greatly reduces particle tracking accuracy. In this report, we demonstrate the severity of these errors by tracking experimental (and simulated) imaging data of optically trapped silver nanoparticles and single fluorescent proteins. We show that errors in interparticle separation, angle and mean square displacement are significantly reduced by applying the corrective Single-Pixel Interior Filling Function (SPIFF) algorithm. Our work demonstrates the potential ubiquity of such errors and the general applicability of SPIFF correction to many experimental fields.

## Introduction

Imaging has become an increasingly important part of scientific research. Breakthroughs in biology^1^, material science^2,3^ and astrophysics^4^ have been made possible by advances in imaging systems^5^ and techniques that allow rapid measurement of physical phenomena below the resolution limit^6–9^. In an optical imaging system, light from an object of interest is focused onto a detector and converted to electrons that are digitized and stored as a two-dimensional array (of pixels) representing the position-dependent intensity map in space (and a third dimension as a video, in time). Once the image is obtained, mathematical algorithms are used to determine the particle positions in each frame, and to track them. One needs to establish the unique identities of the individual particles across frames, to create trajectories.

Many particle tracking algorithms use the distribution of pixel intensities along with knowledge about the point-spread function (PSF) of the system to localize the particle with sub-pixel accuracy. Widely used approaches include: the “Crocker-Grier” algorithm^10^, which determines the “center of mass” of pixel intensities to estimate the location of the particle; the Raghuveer algorithm^11^, which calculates the maximum radial gradient around the particle to estimate its center; and non-linear fitting of a Gaussian function to the pixel intensity distribution^12,13^. However, despite continued development of algorithms and benchmark comparisons between them^14–16^, problems still arise with their accuracy and efficacy, especially when images suffer from low signal-to-noise, are cluttered with multiple objects in close proximity, or are of particularly small objects.

One such problem is that most algorithms, to a greater or lesser degree, bias the sub-pixel location of a tracked object towards the center of the calculated pixel. This effect is known as “pixel locking” or “pixel biasing”^17–19^. Recently, Burov *et al*.^20^ reported the Single Pixel Interior Fill Function (SPIFF) algorithm that corrects this bias even in cases where the actual tracking is done with a proprietary method and is unknown. The correction of the pixel locking error is done by analyzing a set of images, collecting the fractional part of the tracked locations in a “meta-pixel”, and determining if the resultant distribution is uniform within the meta-pixel. The SPIFF algorithm uses this distribution to estimate the true particle position by expanding the probability distribution such that it uniformly spans the entire meta-pixel. Thus, given a set of estimated sub-pixel values of particle centers $\{{\hat{X}}_{E}\}$, the true particle position, ${\hat{X}}_{T}$, can be calculated by numerically solving the integral^20^

1 $${\hat{X}}_{T}=\pm {\int }_{0}^{{\hat{X}}_{E}}P({\hat{X}}_{E}^{\text{'}})d{\hat{X}}_{E}^{\text{'}},$$

and finding the fraction of estimated values in the range $(0,{\hat{X}}_{E})$, where $P({\hat{X}}_{E}^{\text{'}})$ is the SPIFF density function, i.e. the probability density of the set $\{{\hat{X}}_{E}\}$ (see ref.^20^ and Supporting Information).

In this report we establish the importance of the SPIFF algorithm by demonstrating the prevalence of pixel locking errors in common experimental systems (beyond the colloidal particles considered in^20^), their detrimental effect on experimental outcomes, and how they can be identified and corrected. We study tracking errors of nanoparticles and single molecules and demonstrate quantitatively the consequences of the error correction that the SPIFF algorithm provides vis-a-vis its effect on statistical and dynamical properties of these systems. We demonstrate the severity of errors that can arise from biased tracking for common experimental conditions when the size of the particles (and/or the tracking window) is small (i.e. occupies two-square to four-square pixels on the detector), and the sampling rate is high (i.e. particle motion is sub-pixel per frame). We also illustrate how the SPIFF algorithm ameliorates these errors and improves the fidelity of experimentally extracted quantities such as particle trajectories or mean square displacement (MSD). We also show limitations of a “global” SPIFF correction and offer additional strategies for 2^nd^ tier error correction in the Supporting Information. Overall, we demonstrate pixel locking error resulting from common biases inherent to a wide range of experimental measurements can be easily identified and corrected.

## Results

### Nanoparticle imaging

Since nanoparticles are often smaller than the PSF of optical microscopes and thus can be separated by distances less than the resolution limit, errors in their localization can be severe. Therefore, we considered the general problem of tracking several closely spaced nanoparticles. We analyze experimental data obtained by imaging 150 nm diameter Ag nano-particles that are trapped and manipulated using holographic optical tweezers. Our experimental setup is presented in the Supporting Information. Optical tweezers have been used in a wide range of research fields as they allow the manipulation of micro and nanometer sized objects^21,22^ in order to understand interparticle dynamics and behavior^22^ or light-matter interactions^23^. The goal of our experiment is to understand the forces and torques exerted on collections of nanoparticles for different polarization states of the trapping beam. Therefore, extracting accurate interparticle separations and angles from tracking particle locations is crucial.

**Figure 1:**
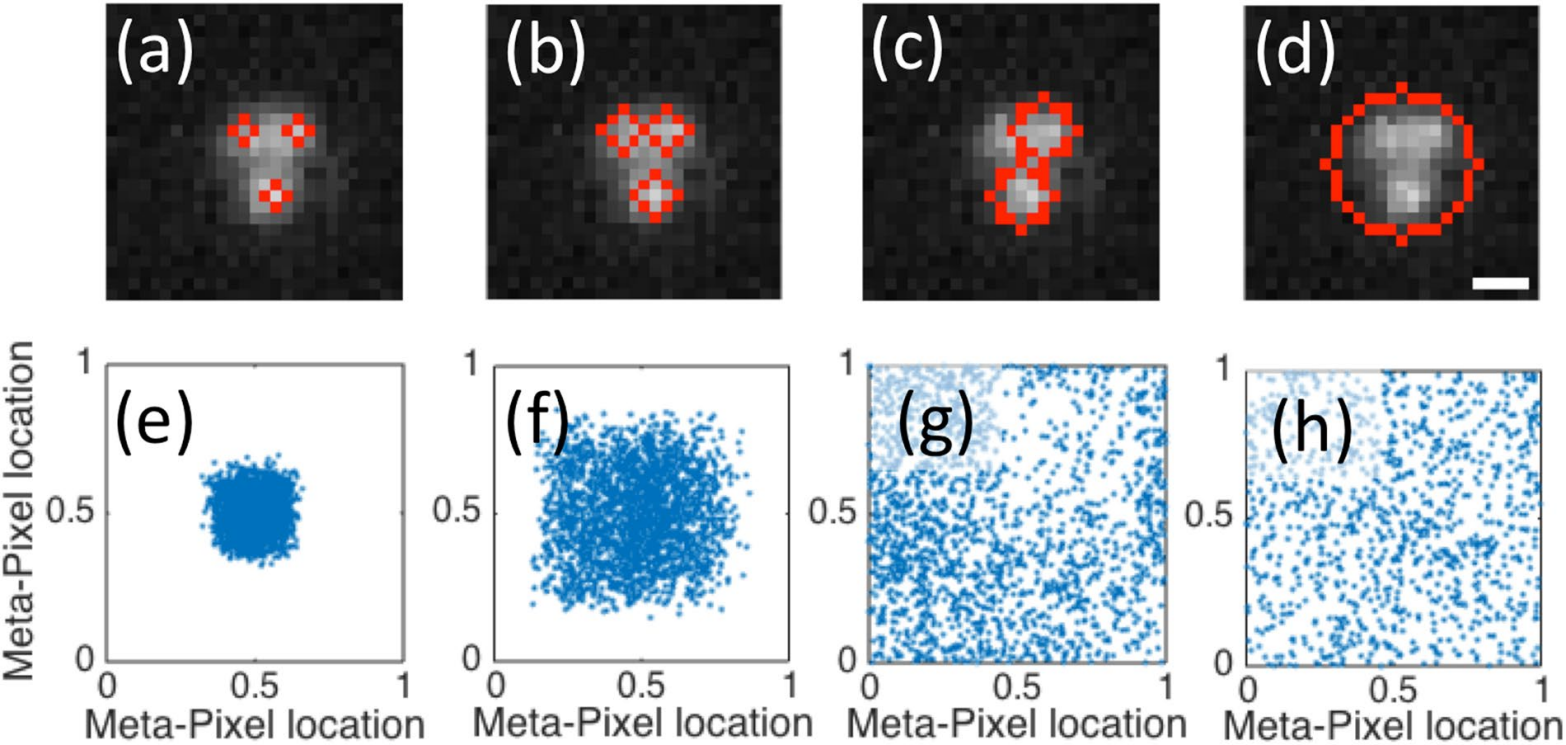
The effect of window size, *W*, or windowing on the particle identification (top row) and the associated meta-pixel distributions (bottom row). Images (**a**–**d**) show a representative frame taken from a video of three 150 nm diameter Ag nanoparticles trapped in a linearly polarized Gaussian trap. The scale bar represents 360 nm. The particles in the frame were identified by running the Mosaic algorithm described in the text with window radius values of 1 pixel (frame **a**), 2 pixels (**b**), 3 pixels (**c**) and 7 pixels (**d**). Panels e-h show the corresponding sub-pixel localization distributions obtained from analyzing 10^3^ frames of a video of 3 nanoparticles in an optical trap. The experimental magnification of 90x gives an effective pixel size of 72 × 72 nm.

As shown in Fig. 1, which is a representative frame taken from such an experiment, two or more particles can be confined in the electrodynamic near-field, i.e. they are separated by roughly 200 nm center-to-center making particle tracking difficult due to the overlap of the individual particle images on the detector. The size of the nanoparticles and their proximity necessitates that the window function used for tracking only be a few pixels wide to avoid misidentification of 2 particles as a single one. Such small widows manifest under-sampling errors and cause strong pixel locking^20^. To reduce or avoid pixel locking, one can increase the size of the tracking window, *W,* used for fitting to the (expected) Gaussian intensity distribution. This could indeed work when the particles are large (compared to the PSF) or are well separated, but when small particles are in close proximity their images overlap. Thus, the particle center locations are necessarily in error, and an increasing fraction of the particles are misidentified. Misidentification (and undercounting) is not an error that can be corrected by the SPIFF algorithm. More specialized methods that model multiple particles with prior information might be used instead^24^. Herein, we deal with pixel locking error and SPIFF correction.

The tradeoff between tracking window size and misidentification is demonstrated in Fig. 1, where 1000 frames taken from an experimental video of the Ag nanoparticles were tracked using the Mosaic tracking software for ImageJ^25^, which uses nonlinear least squares Gaussian fitting for centroid determination. In Fig. 1 we used “circular” windows with radii *R* = 1, 2, 3  and 7 pixels (window size *W* is defined as *W* = *2R* + *1*). Row 1(a–d) in the figure shows representative images of the localization for each window size while row 1(e–h) shows the corresponding distributions of sub-pixel localizations in the meta-pixel. In addition, we counted the number of particles found for each window radius and calculated the percentage of frames in which 3 particles were identified to be 93%, 83%, 58% and 33%, respectively. The compression of the density toward the center of the meta-pixel in Fig. 1e,f is (a signature of) pixel locking error created by a small window; i.e., a Nyquist sampling error or bias^20^.

When a small window size is used, almost all the particles are identified and tracked in each frame, but the pixel locking error is pronounced and any metric obtained from the particle tracking (distance, angle, MSD etc.) will be biased. The errors are less pronounced for larger window sizes, but at the cost of misidentifying (and losing) particles. Taken ad absurdum, we reach the results shown in Fig. 1d,h; when the window is large enough to encompass all three particles, the tracking algorithm treats them as a single entity and the pixel locking error vanishes. However, the tracked “particle” clearly has little to do with the actual experimental results.

The magnification of the optical system directly determines the size of the particles on the detector (i.e. the number of in pixels it occupies on the detector) as it determines the effective pixel size of the system. Therefore, it could be assumed that increasing the magnification would allow for a smaller effective pixel and a larger image of the particle, which in turn allows one to increase the size of the tracking window and to circumvent the issue of pixel locking. However, increasing the magnification spreads the photons from the imaged object among more pixels, thereby reducing the signal-to-noise ratio (SNR) of the system. This is of particular concern in the case of photon-starved applications such as high frame rate videos or imaging of single fluorescent particles. An analysis of the behavior of our imaging system and SPIFF correction under different magnifications is given in section 2 of the Supporting Information, in which we compare the SNR of our optical system for different magnification values (60x, 90x, 150x, 225x). We find that the SNR of the system decreases with greater magnification. As a result, the accuracy of particle localization algorithms is reduced, despite the use of a larger tracking window and the avoidance of pixel locking. Therefore, we find that the combination of a larger effective pixel size (and higher SNR) with SPIFF correction gives more accurate localization results than a smaller effective pixel size with a lower SNR (see Figs S2–S4). For the experiments presented here, magnification of 90x is suitable for imaging the scattered light from the Ag nanoparticle in accordance to the guidelines given in ref.^26^.

### Tracking Synthetic experimental data

**Figure 2:**
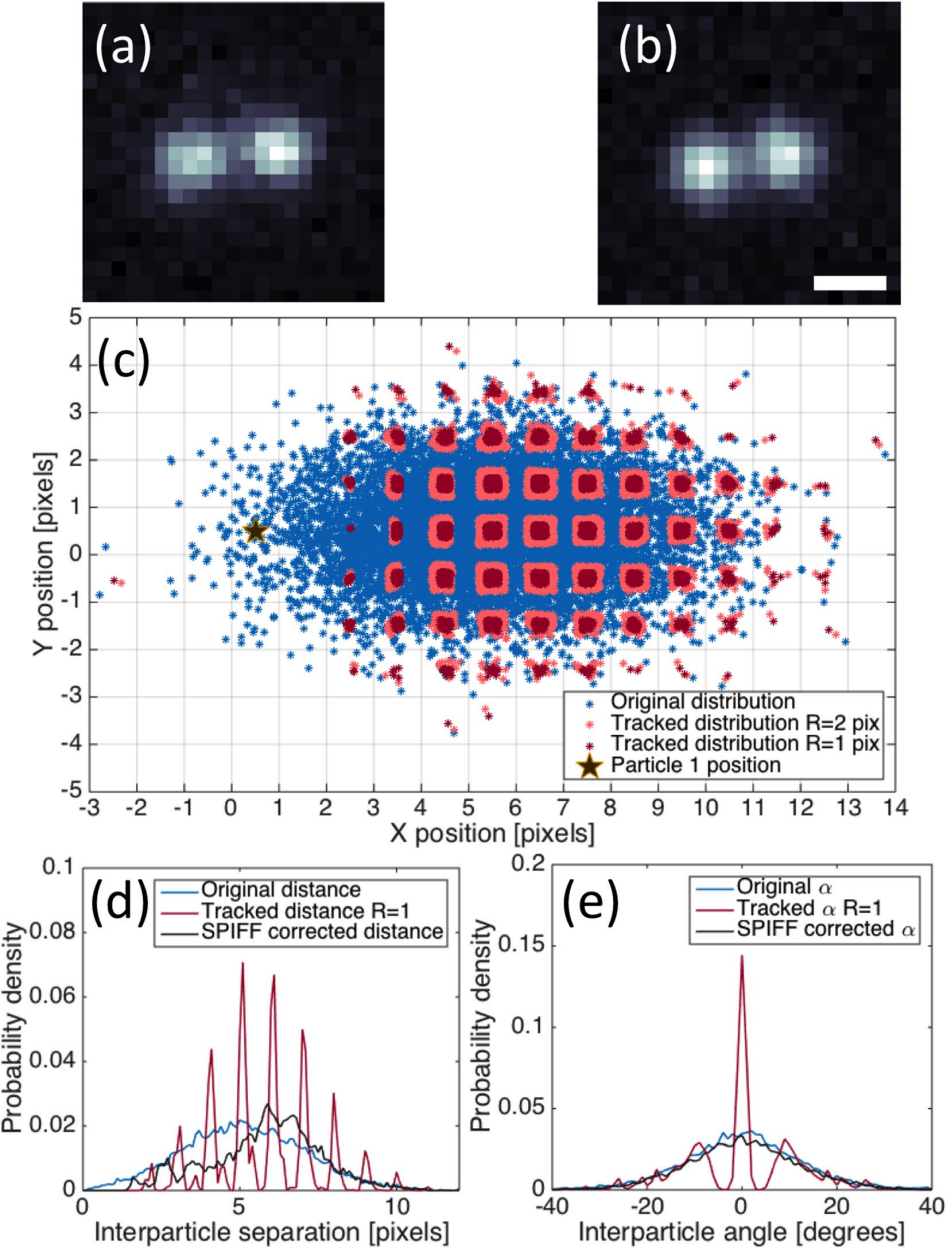
Representative examples of experimental (**a**) and synthetic (**b**) images of two particles separated by 440 nm. Scale bar represents 360 nm. Details of the experimental setup and procedure for creating synthetic images are described in main text and Supporting Information. (**c**) Distribution maps for particle 1 (marked as black star) and true positions for particle 2 (blue dots). The associated synthetic images were tracked using the Mosaic nonlinear least-square Gaussian fitting algorithm as described in the main text. The red and pink dots show the distributions of tracked particle positions with windowing radii of R = 1 (red) and R = 2 (pink), respectively. Note that particle 1 was positioned at (0.5, 0.5) which is the center of pixel (0,0), the definition of a pixel location in the Mosaic algorithm. (**d**) Probability density functions of Cartesian interparticle separation and (**e**) angle for the original data (blue) as well as the tracking algorithm biased (red) and SPIFF corrected values (black). Only frames in which both particles are identified are considered in the distribution map and the probability density histograms. As a result, there are fewer measurements in the tracked and SPIFF corrected distributions in panels (**c**,**d**) and these distributions are normalized relative to the true particle count. The normalization is done to avoid artificially boosting the probability distribution values at larger separations where both particles are consistently identified.

Since it is impossible to know the “true” positions of the tracked particles in an optical trapping experiment of mobile particles, we simulated the results of such an experiment and applied the tracking algorithm to create a benchmark for the SPIFF correction efficacy. This was achieved by simulating frames of two silver particles at varying separations based on experimental videos (such as that described in^22^). We used typical background intensity and variation and particle intensity and size to create frames of synthetic data (images) that simulate the intensity distribution on the detector for given particle locations. The procedure for image synthesis is given in the Supporting Information. Representative examples of experimental and synthetic images of two 150 nm Ag particles are shown in Fig. 2a,b. Similar procedures have been used in the past to compare tracking algorithms^15^ and to assess tracking errors^27^.

Using this method, we synthesized images of nanoparticles at arbitrary separations and investigated the results of particle tracking. The procedure to create synthetic data was applied to a list of 10^4^ positions in which the first particle was fixed at the center of pixel (0,0) while the second one was randomly positioned around (5,0) according to a normal distribution with standard deviations *σ*_*x*_ = *2* and *σ*_*y*_ = 1 pixels (see Supporting Information). For each frame, we tracked the positions of the particle using the Mosaic algorithm with window radii of *R* = 1 (essentially a Swiss cross shaped particle identification window), and 2. The tradeoff described earlier was inherent in this analysis as the Mosaic algorithm correctly identified two particles in 84% of all frames when we used a window with *R* = *1*, compared to 79% of all frames with *R* = 2.

The pixel locking bias is evident in Fig. 2c, which shows the original distribution of particle 2 as well as the tracked positions for all frames where two particles were identified (neglecting cases where the particles were too close to be visually separated). As the tracking algorithm locks the particle location towards the center of a pixel, the distribution of particle positions changes from a Gaussian (blue dots) into sub-pixel regions on a two-dimensional lattice (red and pink sub-pixel size squares). It is evident from this figure that a smaller tracking window compresses the tracked distribution towards the center of the meta-pixel, as shown in Fig. 1. In addition, note the absence of pink dots in the areas where the interparticle separation was small. The fact that only red dots (*R* = *1)* are observed for those small separations means that the algorithm only succeeds in identifying two particles for the smaller value of *R*.

We corrected the pixel locking bias using the SPIFF algorithm given by Eqn. (1). Figure 2d,e shows the interparticle angles and separations, two metrics commonly used in particle dynamics analysis, generated from the tracked and corrected localizations rate. As evident from Fig. 2d, the calculated interparticle separation is strongly affected by the bias caused by small window sizes due to a Nyquist sampling error. Since the tracked positions are locked to the centers of the pixels, the calculated Cartesian separation becomes discontinuous, evident as red spikes. The SPIFF corrected localizations, represented by the black distributions in the panels, alleviates the pixel locking error. The correction works better for larger separations than for smaller ones. One reason for this is that the intensity distributions overlap more strongly for small separations, thereby decreasing the accuracy of centroid determination^20^. Additionally, the dearth of points at smaller separations means that the correction obtained from solving Eqn. (1) underrepresents these points and is skewed. We discuss these issues in the Supporting Information.

The calculated interparticle angle, presented as the red distribution in Fig. 2e, shows another striking error. The angles around ± 5° are “forbidden” owing to the discontinuous nature of the pixel locked particle positions. These angular errors are corrected by applying the SPIFF algorithm to the tracked results; the correction is evident by comparing the blue and black distributions that represent the original and SPIFF-corrected interparticle angles, respectively. The improvement in the distribution of angles is better than for the distribution of separations because the SPIFF correction “fills in” all the discontinuities, thereby recovering values of angles that were missing in the original tracking distribution. By contrast, while the SPIFF correction improves the distribution of interparticle separations, it cannot recover instances when the particles are too close together and are not individually identified. Thus, most of the frames in which the interparticle separation is smaller than roughly 5 pixels (i.e. ~360 nm), are not identified, and are therefore unrepresented in the distribution in Fig. 2d. It is worth noting that while the metrics we analyze here are dependent on two particles being tracked, the SPIFF algorithm is not only applicable to experiments with two particles. Since the algorithm corrects the position of each tracked particle independently, it can improve the results obtained from experiments with any number of tracked particles. This is shown later in this paper for a single particle trajectory (such as in the case of MSD – see section *Analysis of simulated particle trajectories*) and for more than two particles (three particles, see  section *SPIFF correction of experimental data*).

### Analysis of simulated particle trajectories

Next, we analyze the results from a physically realistic simulation based on a combination of finite difference time domain (FDTD) electrodynamics simulation with Langevin dynamics (termed ED-LD). These particle trajectory data from ED-LD simulations allow analyzing the bias and SPIFF correction when the probability distribution of particle positions, $P({\hat{X}}_{E}^{\text{'}}),$ matches experimental results^28^. The ED-LD simulation data also allows exploring the MSD of a particle. We simulated the trajectories of two 150 nm diameter Ag particles trapped in a focused Gaussian beam linearly polarized along the x axis using methodology and parameters described previously^28^. We synthesized images of the particles at these simulated positions that were then tracked. The resultant trajectories consist of 3,200 frames with a time step of 0.5 *μs*. This time step is significantly smaller than what is obtainable by experimental means. However, it is useful as it allows us to gauge the behavior of the tracking algorithm and SPIFF correction when the particle displacement per frame is sub-pixel.

**Figure 3:**
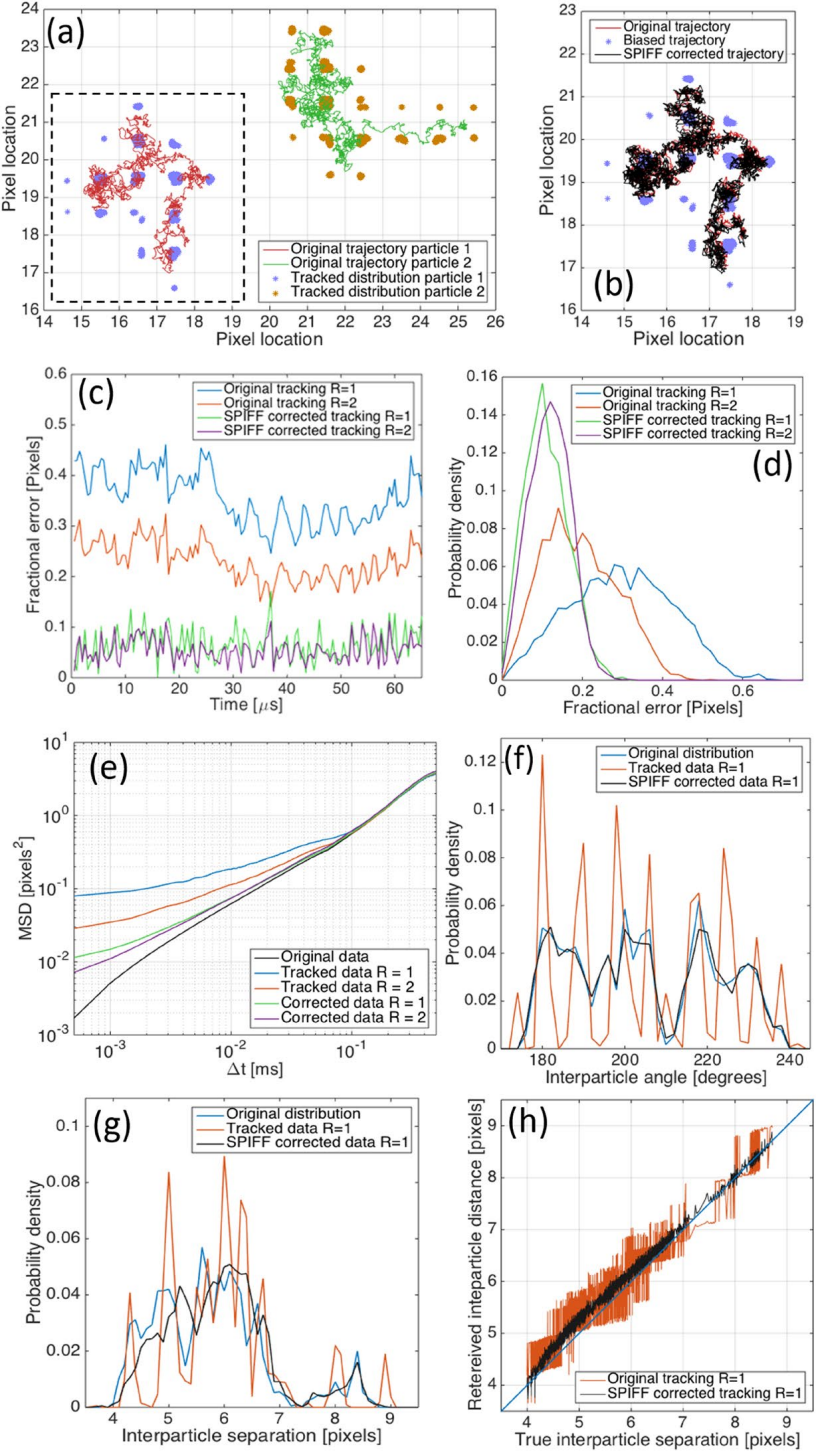
Results from tracking analysis ED-LD simulations of Ag nanoparticle pairs. Images synthesized at the positions obtained from the simulation described in main text and Supporting Information. (**a**) Original particle trajectories for particles 1 (red) and 2 (green) from simulations. The associated localizations from tracking the synthesized images using the Mosaic program with a window of radius *R* = 1 (blue, orange dots) exhibit severe pixel locking. Pixel size is 72 nm. (**b**) The trajectory recovered for particle 1 (dashed rectangular box in panel (**a**)). Red connected points are the original particle trajectory, blue dots are the tracked positions and black connected points are the trajectory obtained after SPIFF correction. (**c**) RMS trajectory error for the first 150 frames of the simulation. The results shown are the RMS error for frames tracked with the Mosaic algorithm and a window of radii *R* = 1, 2 before and after SPIFF correction. (**d**) RMS error distribution for the entire tracked trajectory shown in panel (**b**). (**e**) Logarithmic plot of mean square displacement (MSD) of particle 1 as a function of time step Δt (i.e. inverse frame rate) for original, tracked and corrected trajectories. Calculated interparticle angle (**f**) and separation (**g**) probability densities (using a window of *R* = 1) and the SPIFF corrected data (black). Note that the accuracy of the separation calculation is reduced for smaller separations as explained in the main text. (**h**) Retreived  interparticle separation for the tracked (red) and SPIFF corrected (black) trajectories as a function of the true interparticle separation. Blue line is x = y.

Figure 3 shows the trajectories of two electrodynamically interacting particles and the analysis of the effects of tracking and SPIFF correction (video given in Supporting Information). Figure 3b shows the original trajectory of the particle on the left (in red) as well as its tracked positions, which are the blue dots that are biased towards the pixel centers (i.e. the particle localizations are restricted to the centers of a few pixels). It is clear from the biased distribution that much information about the actual particle dynamics is lost. By contrast, the SPIFF-corrected trajectory (black) in Fig. 3b is a high-fidelity reconstruction of the original particle path. The fidelity of the SPIFF-corrected data is further demonstrated in Fig. 3c,d, which shows the RMS error, defined as the square root of the Cartesian difference between the particle’s true position and its tracked position, before (blue and red) or after (green and purple) SPIFF correction. Figure 3c shows the time evolution of this error throughout the first 150 frames of the simulation and Fig. 3d shows their associated probability densities over the entire simulation. The mean error for the tracked data (using the Mosaic program) are 0.3 and 0.19 pixels with standard deviations of 0.13 and 0.09 for windows of radius *R* = 1 or 2, respectively. After applying the SPIFF correction algorithm this was improved to 0.11 with a standard deviation of 0.05 pixels for both tracking window sizes (i.e. *R* = 1, 2).

The SPIFF correction algorithm allows more accurate measurement of physically significant properties such as the mean square displacement (MSD) of a particle, defined as:

2 $$MSD(\tau )=\overline{{(\vec{x}(t+\tau )-\vec{x}(\tau ))}^{2}},$$

where $\vec{x}(\tau )$is the position of a particle at a time $\tau$ and $\vec{x}(t+\tau )$ its position after time *t*. This measure is used to ascertain the nature of particle motion in conjunction with particle tracking algorithms for a wide range of fields including biophysics^29^ and fluid dynamics^30^ where the statistical mechanical properties of diffusing and driven particles are of interest. The strength of the SPIFF correction algorithm is evident in Fig. 3e, which shows that the error of the SPIFF corrected MSD is reduced by almost an order of magnitude relative to the tracked data, increasing the fidelity of the measured results to the original trajectory and dynamics. As has been shown in the past^17,27^, MSD calculations at short lag times are often susceptible to errors from causes such as particle streaking due to finite exposure time. It is worth noting that apparent super-diffusive behavior was reported in^27^ is in contrast to the apparent sub-diffusive behavior seen in Fig. 3e and in the work by Burov *et al*.^20^

Analysis of interparticle separation and angle are shown in Fig. 3f,g. Similar to Fig. 2, we observe significant errors, resulting from discontinuities in the probability distributions that are ameliorated by the SPIFF correction. The correction is better at larger interparticle separations (>5 pixels) than at smaller ones because, as explained previously, the tracking algorithm has difficulty identifying two particles at small separations, and the meta-pixel distribution is increasingly skewed from the pixel center as the separation decreases. This is evident in Fig. 3h, which shows the relationship between the true interparticle separation (black connected dots) and the separation calculated from both the tracked (red) and the SPIFF corrected data (black, using a window with *R* = 1). As shown in the panel, the error in the calculated separation is not constant but varies with true interparticle separation. This separation-dependent  error can be further used to improve the accuracy of interparticle separation calculations. A simple method to achieving this is by fitting the error curve shown in Fig. 3h to a cubic function and using the resulting fit as a second correction step to improve the SPIFF corrected interparticle separation. This idea is discussed in full in the Supporting Information and is shown to further improve the accuracy of the separation results, especially when the interparticle separation is small.

### SPIFF correction of experimental data

**Figure 4:**
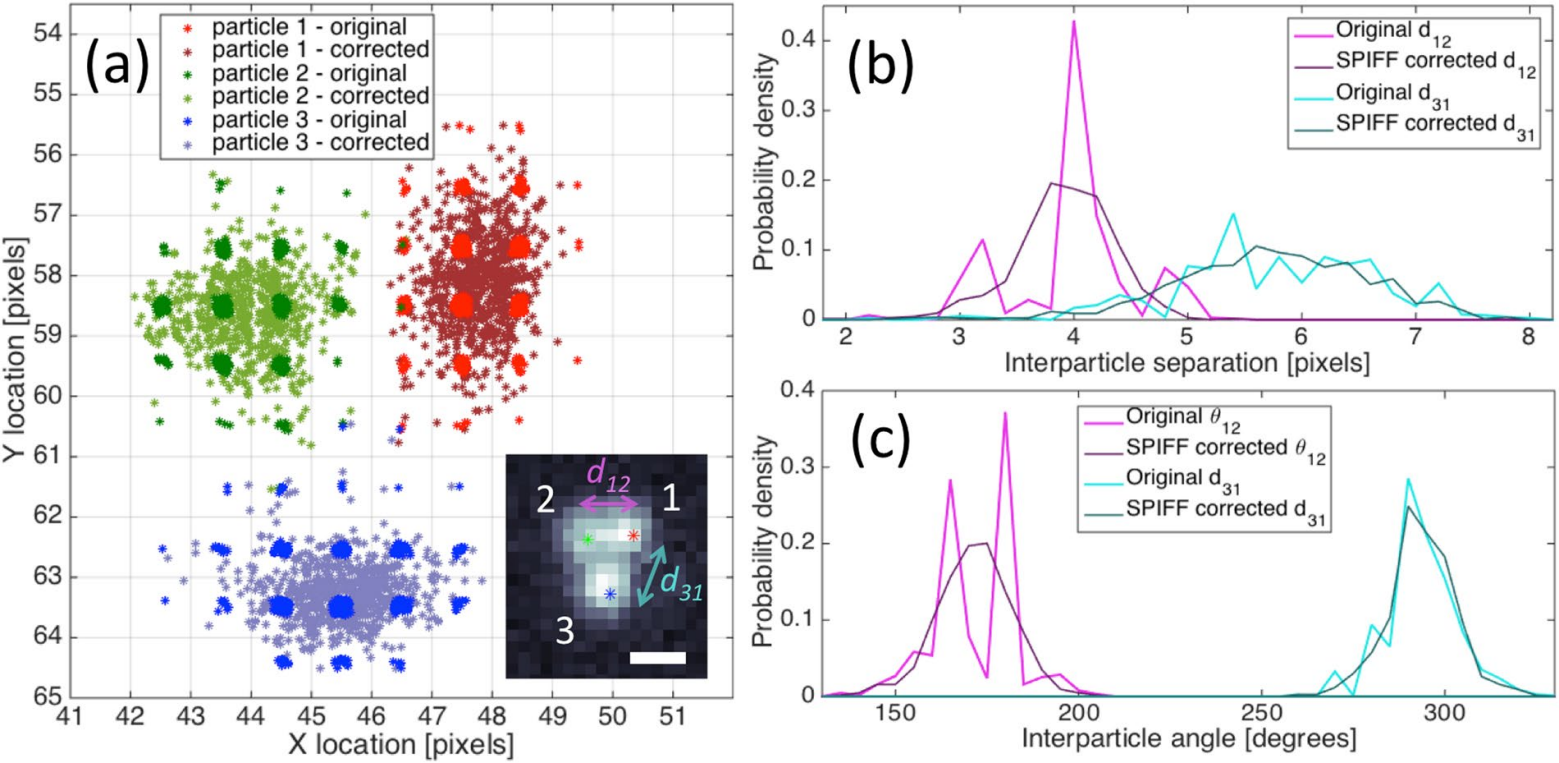
Analysis of an experimental video of three particles trapped in a linearly polarized Gaussian trap. Pixel dimensions are 72 nm. (**a**) Particle localizations from tracking before and after SPIFF correction for the three particles. The red, green and blue marked particles are defined as particles 1, 2 and 3, respectively. Inset - Representative frame from the video (full video given in SI, scale bar represents 360 nm). Interparticle separation (**b**), and angle (**c**) for the three particles. The lighter and darker connected points represent the original and tracked SPIFF-corrected values, respectively. Note the differences in the shapes of the distribution before SPIFF correction between the near-field bound particles (magenta connected points - d_12_) and the optically bound ones (cyan - d_31_).

With these insights from simulated data, we applied the SPIFF correction algorithm to experimental data in which three Ag particles were trapped in a linearly polarized Gaussian trap. Due to the tightness of the trap and electromagnetic interactions between the particles, two particles were trapped in close (near-field) proximity near the center of the focused optical beam with a center–to-center separation of approximately 250 nm (~3–4 pixels), while the third particle was trapped close to the optical binding distance^22,31,32^ with a center-to-center separation of around 470 nm (~6–7 pixels). The particles were trapped using the same laser beam, and any fluctuations in the laser relative intensity^33,34^ or position due to changes in the laser or the optical setup^35^ might cause them to move in a correlated fashion. Regardless, this should not affect the particle tracking and SPIFF correction. We captured images at 1000FPS and analyzed 780 frames. Only frames where all three particles were identified were taken into consideration (see video S2). Figure 4 shows a representative frame from the video as well as the distributions of particle localizations obtained using the Mosaic program with an *R* = 1 window. The distribution of tracked particle positions exhibit significant pixel-locking.

Figure 4b,c shows the same errors that are apparent in our previous analyses of particles at small separations. The distribution of interparticle angles, *θ*_12_ (magenta curve), exhibits a similar angular profile to those observed in the simulated data in Figs 2e and 3g. The omission of some angles for particles in close proximity is entirely the result of pixel-locking. The SPIFF correction recovers a continuous angular distribution as is appropriate for Brownian motion. The simulated errors are also observed for the interparticle distance *d*_12_. Note that the distribution of errors in angle and separation are less prominent, and the SPIFF correction is milder, for particles with larger separations, such as *d*_13_ (cyan curves). However, pixel-locking exists even for particle 3 as shown in Fig. 4a. Despite the relative short length of the time series, the SPIFF correction works well due to the fact that the meta-pixel is composed of the aggregated particle meta-pixel data from all three particles giving roughly 3000 data points. In the Supporting Information we analyze longer experimental videos, which allow for even denser meta-pixels, and compare the tracking results obtained from several widely used algorithms.

### Pixel locking errors and SPIFF correction of single molecule experiments

The determination of the positions and dynamics of single molecules has become an essential experimental method in biophysics and cell biology^36,37^. The small size, low to moderate SNR of the data (i.e. low counts and/or significant background noise) and potentially crowded nature of single molecule images can all result in tracking errors manifest as pixel-locking. We reviewed the data previously published by Lin *et al*.^38^ in which proteins were labeled with fluorophores and imaged as they traveled along flow-extended double-stranded DNA under several flow conditions. The MSD values reported in the paper were obtained from tracking video data using the Gaussian fitting method in DiaTrack 3.0 ^39^ software suite.

**Figure 5:**
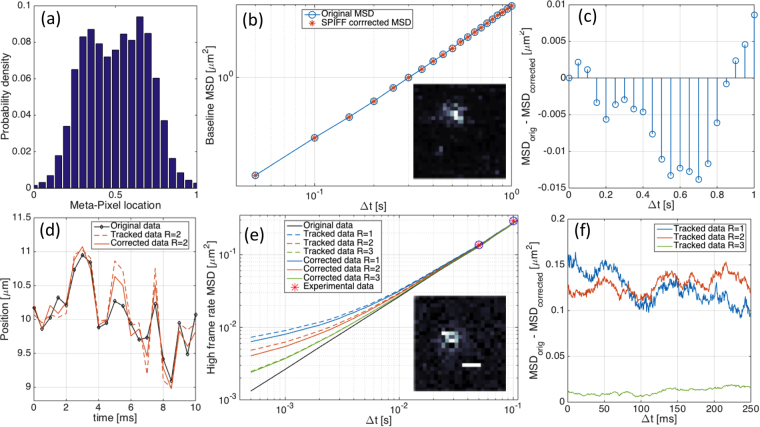
Analysis of tracking data of single molecules moving along a flow-extended ds-DNA as explained in the text. (**a**) Sub-pixel localization distributions obtained from the x-coordinate of the tracked single molecule positions. The non-uniformity of the distribution is pixel-locking error. (**b**) Original and SPIFF corrected MSD values based on trajectories reported by Lin *et al*.^38^. Inset shows a representative experimental image. (**c**) Difference between original and SPIFF corrected MSD as a function of time difference. Note that there is no clear trend and the difference is 2 orders of magnitude smaller than the MSD values. (**d**) Original trajectory (connected black points) along with tracked data (tan dashed line) and SPIFF corrected results (tan solid line), from synthesized images with mild intensity filtering. Details of synthetic data generation and the filtering done are given in the Supporting Information. (**e**) MSDs obtained from tracked images that were synthesized from simulated, high frame rate trajectories. For comparison, the pink stars are the first two experimentally measured MSD values shown in panel (**a**) Inset shows representative synthetic image (scale bar represents 400 nm). (**f**) Difference between original and SPIFF-corrected MSD for synthetic data with different radii (*R* = 1, 2, 3). Note that the difference is always positive, implying that the error in the MSD is consistently reduced, and that the values of the differences are significantly greater than in panel (**c**).

We re-analyzed the reported particle trajectories and found pixel-locking in the trajectory data (Fig. 5a). Surprisingly, the SPIFF correction did not improve the results. We attribute this null result to the low frame rate (20FPS) and the associated large displacements of the single molecules in 50 msec. That is, the MSD values obtained (Fig. 5b) are at least two orders of magnitude larger than the pixel locking error (Fig. 5c).

To test this interpretation, we generated synthetic particle trajectories based on the transport properties given by Lin *et al*.^38^, used them to generate particle positions and images at higher frame rates (2000 FPS) and tracked them using Mosaic (see Supporting Information). Similar to the results for nanoparticles, we observe errors in the tracked trajectories and MSD values compared to the true (original) values. As can be seen from Fig. 5d–f these errors are reduced by SPIFF correction, and the improvement is greatest for a window with a radius of *R* = 1. The fact that the SPIFF correction did not reduce the MSD error in the original low frame rate video can be understood as the particle motion being undersampled for efficacious SPIFF correction; i.e. particle displacements between consecutive frames were far larger than the magnitude of the sub-pixel SPIFF corrections. This was confirmed when we reduced the frame rate of our synthesized data (see Supporting Information Figure S15 and section S7).

## Discussion and Conclusions

We have demonstrated the pixel-locking error that is inherent to tracking of nanoscale objects such as nanoparticles, quantum dots and fluorescent single molecules. We showed that this error can be corrected using the SPIFF algorithm, resulting in a marked improvement in the fidelity of the calculated results to the true values. Since the imaging and tracking of nanoscale objects is commonplace in contemporary research, the present work makes clear the ubiquity of tracking errors that can be particularly insidious for such localization studies. The construction of the meta-pixel SPIFF distribution and correction presents a general and straightforward way to identify and correct such errors.

Placed in a broader perspective, SPIFF correction of particle tracking errors is especially valuable when the Nyqiust sampling condition is compromised in both time and space in the experimental data. The SPIFF correction significantly improves the measurement (localization) accuracy when  the tracked particles are small (or when the image is crowded) and the sampling rate is sufficiently large that particle displacement between frames is sub-pixel. Greater magnification cannot simply solve the issue given the diffraction limit and Rayleigh resolution criterion and the tradeoff between precision and accuracy that occurs when spreading a photon-limited source over more pixels (see SI Figures S2–S4). If the data is compromised only in space (i.e. the particles are small or the image  is crowded but the particle motion is not sub-pixel), then the SPIFF algorithm will improve the accuracy of spatial metrics such as interparticle separation or interparticle angle, but will not change the accuracy of time dependent measures such as MSD. This was shown in Fig. 5b,c, where the mean displacement of the fluorescent protein was larger than a single pixel and as a result, the sub-pixel enhancement given by the SPIFF algorithm did not significantly improve the MSD. Finally, if the image data is compromised only in time, i.e. the particles are large or well separated, but the frame rate is insufficient to capture sub-pixel dynamics, then the SPIFF algorithm cannot offer any further improvement to the measured accuracy in neither space nor time since there is no pixel-locking to correct (see SI Figure S15).

Therefore, the importance of this analysis and SPIFF correction will grow as researchers continue to improve the resolution and rate of imaging techniques^40–43^ and demand greater accuracy.

## Electronic supplementary material

Supporting Information

Video S1

Video S2

Video S3

Video S4
