# Supplementary material for: Analysis and correction of errors in nanoscale particle tracking using the Single-pixel interior filling function (SPIFF) algorithm
Source: Sci Rep. 2017 Nov 29;7:16553. doi: 10.1038/s41598-017-14166-6 (PMC5707392; doi:10.1038/s41598-017-14166-6)
Supplement: Supplementary file 1 — Supporting Information [file 41598_2017_14166_MOESM1_ESM.pdf]

Supporting Information for: Analysis and correction of errors in nanoscale particle tracking using the Single-pixel interior filling function (SPIFF) algorithm.

Yuval Yifat[1], Nishant Sule[1], Yihan Lin[3], Norbert F. Scherer[1,2,*]

*[1] James Franck Institute, [2] Department of Chemistry, The University of Chicago, Chicago Il. 60637, USA.*

*[3]Center for Quantitative Biology, Peking-Tsinghua Center for Life Sciences, Academy for Advanced Interdisciplinary Studies, Peking University, Beijing 100871, China.*

* Corresponding Author: nfschere@uchicago.edu

**1. Nanoparticle trapping experimental setup:**

The 150 nm Ag particles described in the text were trapped in a linearly polarized Gaussian laser beam that was focused through a microscope objective. A diagram of the trapping setup along with a representative image (i.e. one frame in a video) from the experiment and measurements of the beam profile and point-spread function (PSF) of a single Ag nanoparticle are shown in Figure S1.

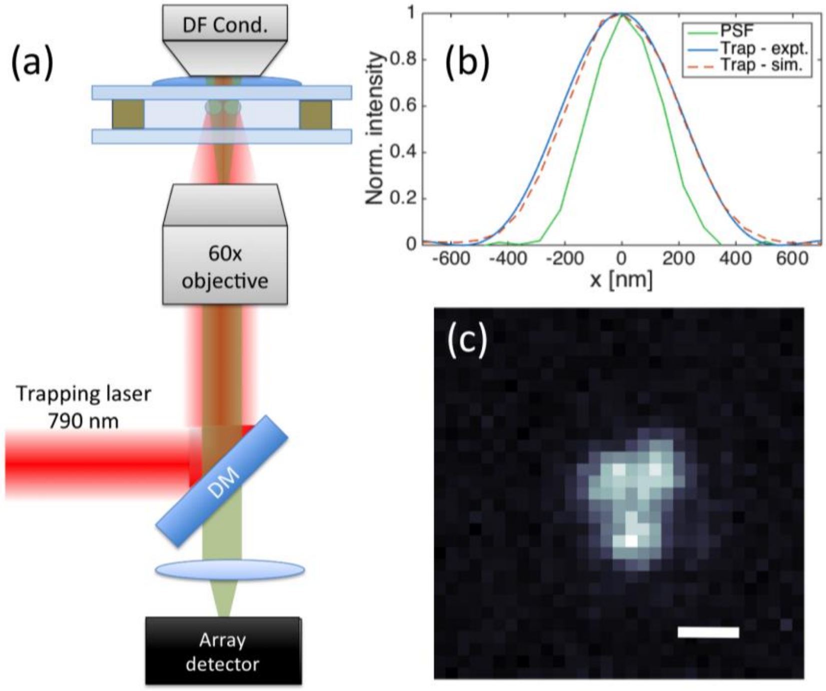


**Figure S1**. (a) Diagram of optical trapping setup used to obtain the experimental data presented in the main text. DF Cond.–Dark field condenser, DM-Dichroic mirror. (b) Plots of the measured intensity of the Gaussian optical trap and the corresponding intensity profile of the focused Gaussian beam used in the ED-LD simulations (allowing measurement of the width of the optical trap). The point-spread function (PSF) of a single Ag nanoparticle for our optical system is also shown. The optical tweezers instrument was used for the optical trapping experiments presented in the text, while the simulated focused Gaussian beam was used for the ED-LD simulations described in Fig. 3 in the main text. The FWHM for both is 450 nm, and the FWHM of the particle PSF is 300 nm. (c) Dark-field scattering image of three Ag particles trapped in the Gaussian laser trap. Scale bar is 360 nm.

The setup consisted of a CW Ti:Sapphire laser emitting linearly polarized (LP) light at a wavelength of 790 nm that was collimated and directed into a Nikon TI inverted optical microscope and through a 60x IR-corrected water immersion objective (Nikon 60x Plan APO IR water immersion objective, NA = 1.27). The incident power was measured to be 8mW before the dichroic mirror. The laser beam was focused into a sample cell that was filled with a solution of 150nm diameter silver nanoparticles coated with a layer of polyvinylpyrrolidone (PVP) diluted in 18MΩ de-ionized water in a ratio of 1:200. The beam was positioned such that its focus would be in the solution slightly below the top cover slip and had a measured FWHM of 450 nm. The particles were illuminated using a dark-field condenser and the light they scattered in a dark-field configuration was captured by the objective and imaged onto an sCMOS camera (Andor Neo; 6.5 µm pixel size) with a total magnification of 90x. The particle motion was captured with an exposure time of 0.1ms at a frame rate of 1040 frames per second. Various magnifications (60x, 90x, 150x, 225x) with combinations of 1.5x internal and 2.5x external magnification optics can be obtained with the setup.

2. **The effect of optical magnification:**

The size of the particles on the CMOS detector, that is the number of pixels they occupy as an image, is due to the optical magnification of the system. If the magnification is too small, the particle images occupy only a few or even a single pixel, increasing the pixel-locking error. Therefore, one might make the claim that increasing the magnification of the system (to 120x, say, as is shown in ref [1] for the same type of particles), would increase the image size on the detector and allow the use of larger windows for particle tracking, thereby decreasing the pixel-locking error. However, increasing the magnification will decrease the SNR of the system as the same number of photons will now be spread over a larger number of pixels, thereby affecting the precision of localization. This is of particular concern in the case of photon-limited experiments such as imaging of single fluorescent molecules or rapidly moving particles that necessitate short integration times.

In order to explore the effect of magnification on the SPIFF algorithm, we trapped a single 150nm diameter Ag nanoparticle in an expanded Gaussian trap using the setup described in section 1. The particle motion was captured at a frame rate of 2004FPS with an exposure time of 100 µs and the videos were 10^4 frames long. These imaging conditions are adequate for measurement of fast dynamics of trapped particles. We also introduced an external 2.5x beam expander to the system allowing us to capture the particle motion with magnifications of 60x, 90x, 150x and 225x, corresponding to effective pixel sizes of 108, 72, 43 and 29 nm, respectively. Figure S2 shows a representative frame from the videos taken for each of the magnifications.

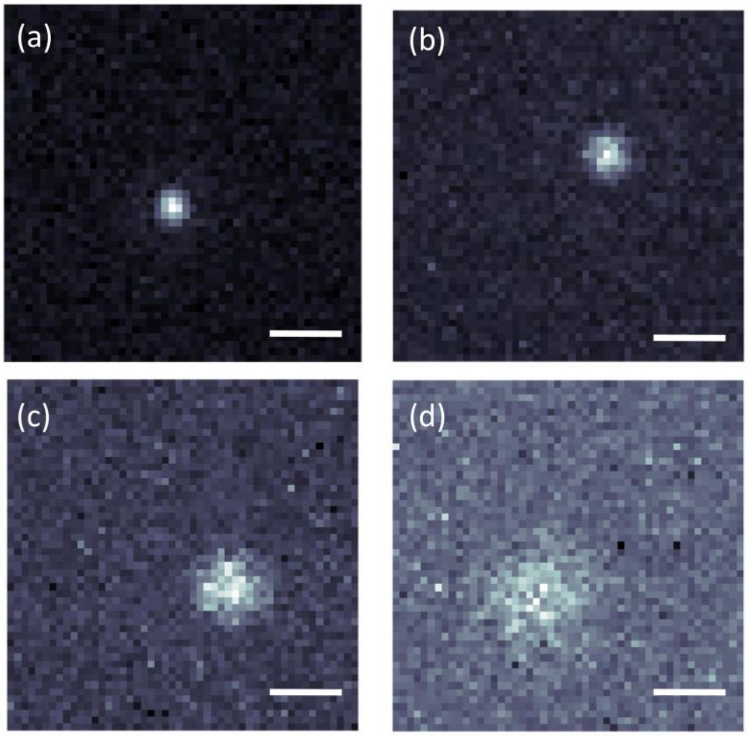


**Figure S2**. Representative dark-field image of a single 150 nm Ag nanoparticle taken at different magnifications. (a) 60x magnification, scale bar is 1080 nm. (b) 90x magnification, scale bar is 720 nm. (c) 150x magnification, scale bar is 430 nm. (d) 225x magnification, scale bar is 288 nm. The illumination intensity was the same for all magnifications

As can be seen from Fig S2, the size of the particle on the detector increases at larger magnifications, but its contrast is reduced (compared to the background). This is due to the fact that the light scattered from the particle is distributed amongst more pixels. We measured the SNR of the videos (defined as the ratio of the brightest pixel in the particles to the mean value of the background pixels) for the different magnifications as 3.3, 2.4, 1.7 and 1.4 for the aforementioned magnifications.

We tracked the particle positions using the Mosaic algorithm with varying window radii and used the tracked trajectories to calculate the mean square displacement (MSD) values for the trapped particle. The results of the MSDs evaluated for different tracking windows and magnifications are shown in Figure S3.

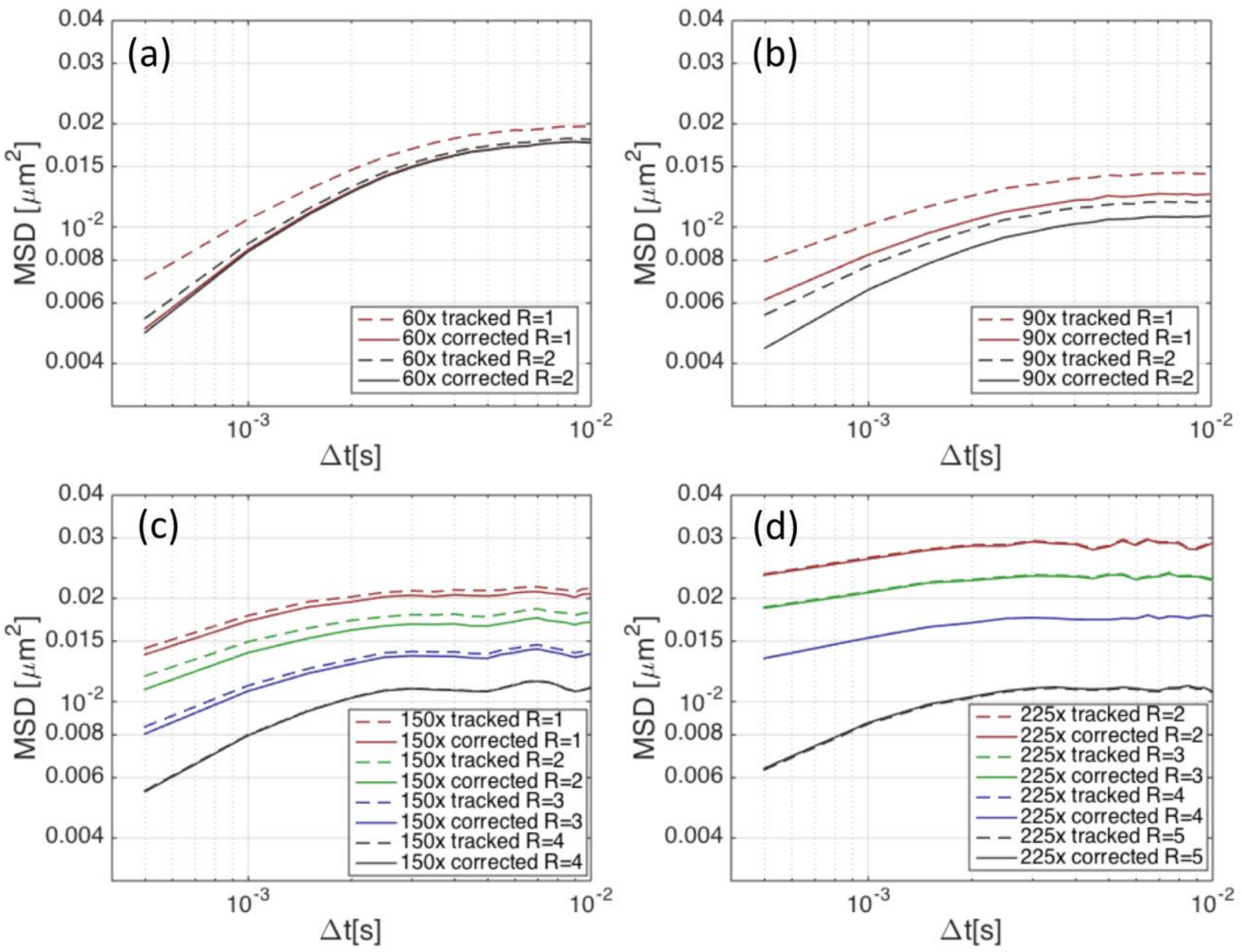


**Figure S3**. Calculated MSD values for a single particle trapped in a focused Gaussian trap for magnifications of (a) 60x, (b) 90x (c) 150x and (d) 225x. The different colored curves represent the different window sizes used by the Mosaic algorithm for particle tracking. The dashed curves are the tracked data, while the solid curves are the results calculated for the same trajectories after SPIFF correction.

Figure S3 compares the MSDs for a single particle trapped under similar conditions but viewed under different magnifications. Thus, the physical behavior of the particle will be the same and any differences in the tracked particle behavior will be due to the differences in the magnification.

When the magnification is small (see Fig. S3(a)), the accuracy of the particle localization is limited by pixel locking effects. As a result, the particle displacement at small time lags (the first step of the MSD) is larger than for the 90x magnification (Fig S3(b)). This error can be improved significantly by using the SPIFF algorithm (compare the dashed and solid curves in Fig S3a).

When the magnification is large (e.g. Fig S4d) we see that the SPIFF algorithm does not significantly improve the MSD, for any tracking window size. For larger windows (e.g. R=5 in panel (d)) this is because the tracked data do not exhibit pixel-locking so the SPIFF algorithm has nothing to correct. For smaller windows (R=2 in panels (c-d)) the localization accuracy is limited by the low SNR of the images, which causes the tracking algorithm to localize the particle position to different pixels and not necessarily on its center. This results in artificially large displacements. These effects are present in both the 150x (Fig S4(c)) and the 225x (Fig S4(d)) magnifications.

Figure S4 compares the MSDs for the largest tracking windows for all magnifications shown in Figure S3.

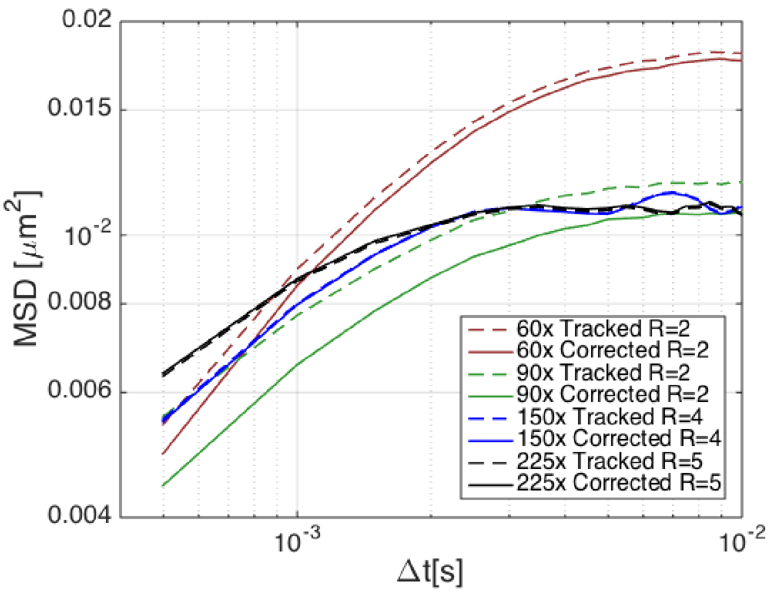


**Figure S4**. MSD values for different magnifications calculated from the tracked data localized using the largest tracking window size.

The curves in Figure S4 show the results obtained from the MSD calculations of each of the magnifications using the largest reasonable tracking window sizes (that is, window sizes that are not larger than the tracked particle). As can be seen, the 90x magnification after particle tracking gives the smallest initial MSD values, implying that this magnification allows the best accuracy and precision in particle localization. This magnification is in accordance with the requirement that the pixel size be roughly 1/2.8 of the diffraction limit of the system[2]. It is important to state that this result is only valid for the optical system and trapping parameters described in section 1. A change in the noise of the system or the particle brightness and size could lead to a different optimal magnification. For example, for brighter particles (and lower detector noise and background levels) greater magnification might be better. More research needs to be done to make a general claim about the optimal conditions for extracting information

about sub-pixel particle positions given a system's SNR and the measured objects brightness and dimensions.

It is worth noting that the improvement of the MSD calculations by the SPIFF correction for the lowest magnification (evident in Figure S3(a) and in the red curves of Fig. S4), is a mark of the SPIFF algorithm restoring some of the information about the particle trajectory lost due to the pixel-locking. Therefore, one could increase the sampling rate of a given experiment without much loss of information by using lower magnification and a smaller field of view, which would allow a higher frame rate, and applying the SPIFF correction to the resulting trajectories.

**3. Preparation and analysis of synthetic images of nanoparticles:**

Since it is not possible to determine the "true" positions of the tracked particles in an optical trapping experiment of mobile particles, we devised a method for simulating the results of such an experiment and applied the tracking algorithms to this "synthetic data", thereby creating a benchmark for the performance of the SPIFF correction. The synthetic image data were created by simulating images of nanoparticles based on actual experimental images and videos (such as those described by Yan et al [3]). We analyzed the images (frames) taken from experimental data and extracted the representative background noise level and its variation, as well as the intensity and width of a typical detected Ag particle (which had a FWHM value of 2 pixels). These values were then used to parameterize a procedure that accepts an input position list $\{x_i, y_i\}_k$ that describes the coordinates of each one of $M$ particles throughout $N$ frames (the dimensions of the list are *2MxN*). We then generate $N$ synthetic frames (images) displaying the positions of the $M$ particles throughout the video. The resulting "synthetic images" (or frames) simulate the intensity distribution that is recorded by a CMOS (or CCD) detector in an experimental setup for a given list of particle positions. An example of experimental and synthesized frames of two 150 nm Ag particles at different separations are shown in Figure S5(a-c) and S5(d-f), respectively.

Once the synthetic images were created, we used the Mosaic tracking program to obtain estimated particle localizations. Also, tracking errors were manifest in interparticle separations and angles as described in Figure 2 in the main text. Specifically, we simulated $10^4$ particle positions of two particles, the first was fixed at location (0,0) while the second was randomly positioned around (5,0) with a Normal distribution with a standard deviation of 2 and 1 pixels along the x and y axes, respectively. A graphical representation of the position distribution of particle 2 for this simulated data is shown in Figure S5(g). The frames, which were synthesized from this distribution, were tracked and analyzed as described in the main text. The data in Fig. S5(g) are the same as shown in Fig. 2(c).

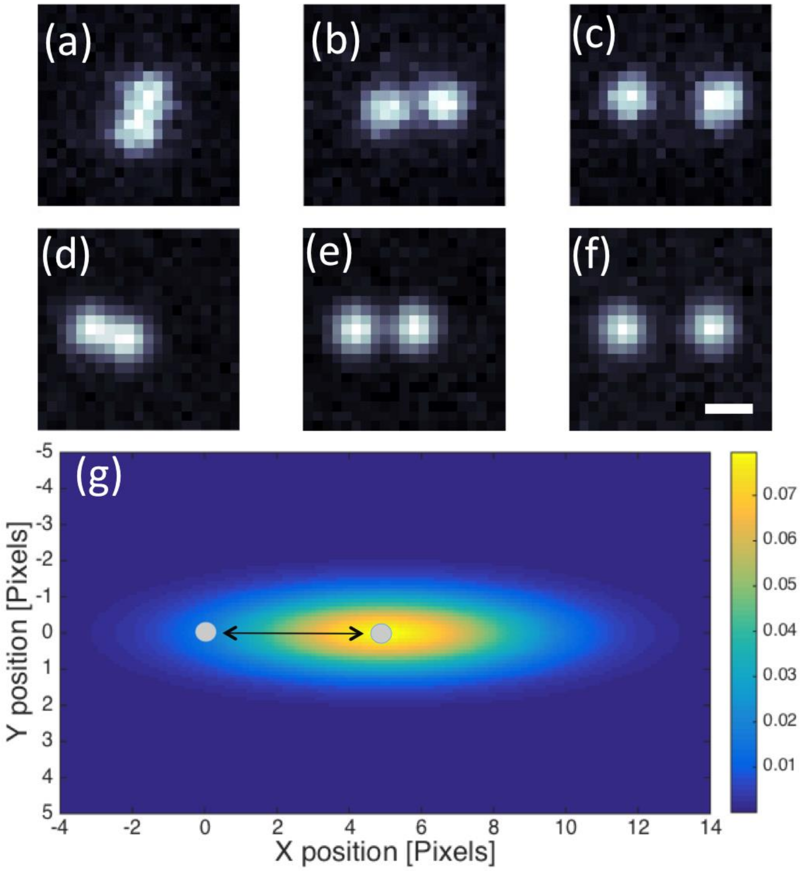


**Figure S5**. Representative experimental images of two 150nm diameter Ag particles separated by (a) 3.5, (b) 6.1, (c) 9.3 pixels respectively. (d-f) Representative synthesized images of similar particles with separations matching the experimental conditions. Scale bar is 360 nm. (g) Probability distribution for the location of particle 2 as described in the main text. Particle 1 is located at (0,0), while particle 2 is placed at $10^4$ positions around (5,0) with a normal distribution with a standard deviation of 2 and 1 pixels along the x and y axes, respectively.

## 4. Comparison between tracking algorithms

To assess the general utility of the SPIFF algorithm, we applied it to the localization results of several widely used, freely available tracking algorithms[4–8]. The algorithms we used were Mosaic for imageJ[4], Trackpy for python[5], Polypixel (polyparticletracker GUI - polynomial fitting with Gaussian weight)[6], Raghuveer (Radial symmetry tracking GUI)[7] and Diatrack Version 3.05[8]. The video we tracked was of two 150nm Ag nanoparticles trapped in a focused left handed circularly polarized Gaussian beam. The specific beam diameter and focusing conditions of the trap created two preferred separations for the particle pair - in the near-field (at around 250 nm center-to-center) and in the optical binding regime (at around 500 nm center-to-center). The video was taken at 1080 FPS with an exposure time of 100μs to reduce particle streaking. Over the course of the video, which consists of $10^4$ frames, the particles

separation varied between the two values, and tracking their position accurately is a crucial step towards constructing a potential mean force (PMF) for the system. Figure S6 shows representative frames of the two particles at three different separations.

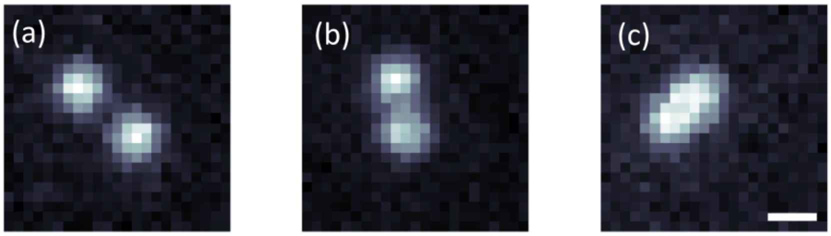


**Figure S6**. Representative experimental images of two 150nm diameter Ag particles trapped in an expanded Gaussian beam. The particle center-to-center spacing was measured to be (a) 550 nm (b) 400 nm (c) 290 nm. Scale bar is 360 nm.

We tracked the particle positions using the algorithms listed below using the following parameters:

1) Mosaic for imageJ[4]: trajectories were obtained using a tracking radius of R=1 pixel, a discrimination cutoff of 2.0 and a percentile brightness threshold (Per/abs) of 0.4. The identification percentage (that is the percentage of frames in which two particles are identified) was 99.4%.

2) Trackpy for Python[5]: Tracking was performed using software version 0.3.2 with a particle diameter of $W=3$ pixels, and a minimal separation of 4 pixels (diameter + 1 pixel). All other parameters were set to their default value. Identification percentage was 88.2%.

3) Polypixel (polyparticletracker GUI - polynomial fitting with Gaussian weight)[6]: We applied both the “Centroid of image” and the “Polynomial Fit Gaussian” methods. The particle radii were set between 1 and 10 pixels and the rest of the parameters were left at their default levels. Identification percentage was 49.1% for the centroid method and 45.9% for the polynomial method.

4) Raghuveer (Radial symmetry tracking)[7]: Tracking was performed using both the radial and the centroid localization methods. The bandpass filter (bpfiltsize) and a particle size (nsize) were both set to 4 pixels (equivalent to $W=4$) and the Intensity threshold was set to 99%. Identification percentage was 83.2% for both the centroid and the radial methods.

5) Diatrack Version 3.05[8]: Tracking was performed after the data was pre-filtered with a value of 0.85. Following that, any tracked objects with a brightness level below 200 were removed. Finally, the high precision was obtained by applying a match to a Gaussian with a FWHM of $R=2$ pixels. Identification percentage was 77%.

Using the trajectories obtained from each one of the algorithms we produced a meta-pixel distribution, and calculated the interparticle separation before and after SPIFF correction. The meta-pixel distributions are shown in Fig. S7.

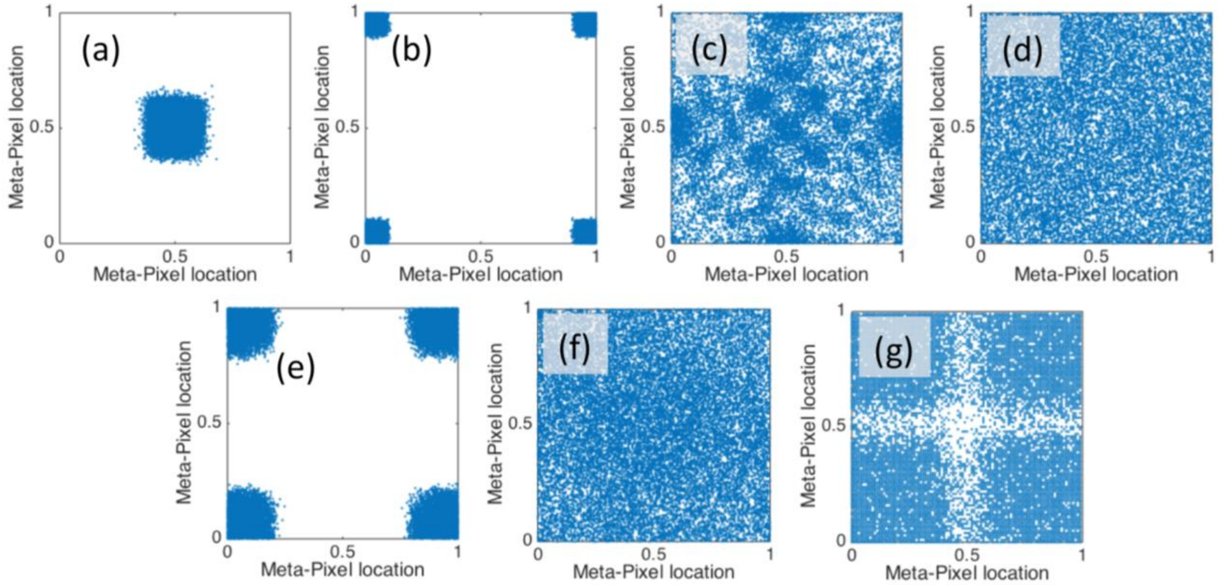


**Figure S7**. The meta-pixel output of the sampled algorithms from tracking the video of two particles trapped in an expanded Gaussian trap. The metapixels shown are for the following aglorithms: (a) Mosaic algorithm for imageJ (b) Trackpy algorithm version 0.3.2 (c) Centroid output from the polypixel algorithm (d) polynomial output from the polypixel algorithm (e) Radial calculation from the Raghuveer software package (f) Centroid calculation from the Raghuveer software package (g) Diatrack 3.05 algorithm.

From comparing the metapixel distributions presented in Fig S7(a-g) it is clear that the problem of pixel locking is not limited to the Mosaic algorithm we used in the main text. Some algorithms show stronger pixel locking than others (e.g. compare (a) to (g)), while some show a different behavior (e.g. panel (c)), and some methods do not display pixel locking. The variety in the metapixel results shows the importance of this analysis for better understanding the behavior and output of different tracking algorithms.

It is important to note that the bias of the particle localization towards the center of the metapixel (Fig. S7(a)) or the corners (Fig. S7(b)) is the result of the implementation of the localization for the different tracking software suites, but is still a manifestation of the same pixel-locking bias. In other words, the difference between panels (a) and (b) is that Mosaic software localizes the particles towards the center of the pixel, while the Trackpy procedure localizes the particles at the pixel edges. These differences necessitate the SPIFF algorithm to compute the probability density relative to a different point (the edge

of the pixel rather then the center) but does not effect on the accuracy of the SPIFF correction. Or one can redefine the center of a pixel to the corner thus collapsing the 4 disconnected regions of pixel density to the center.

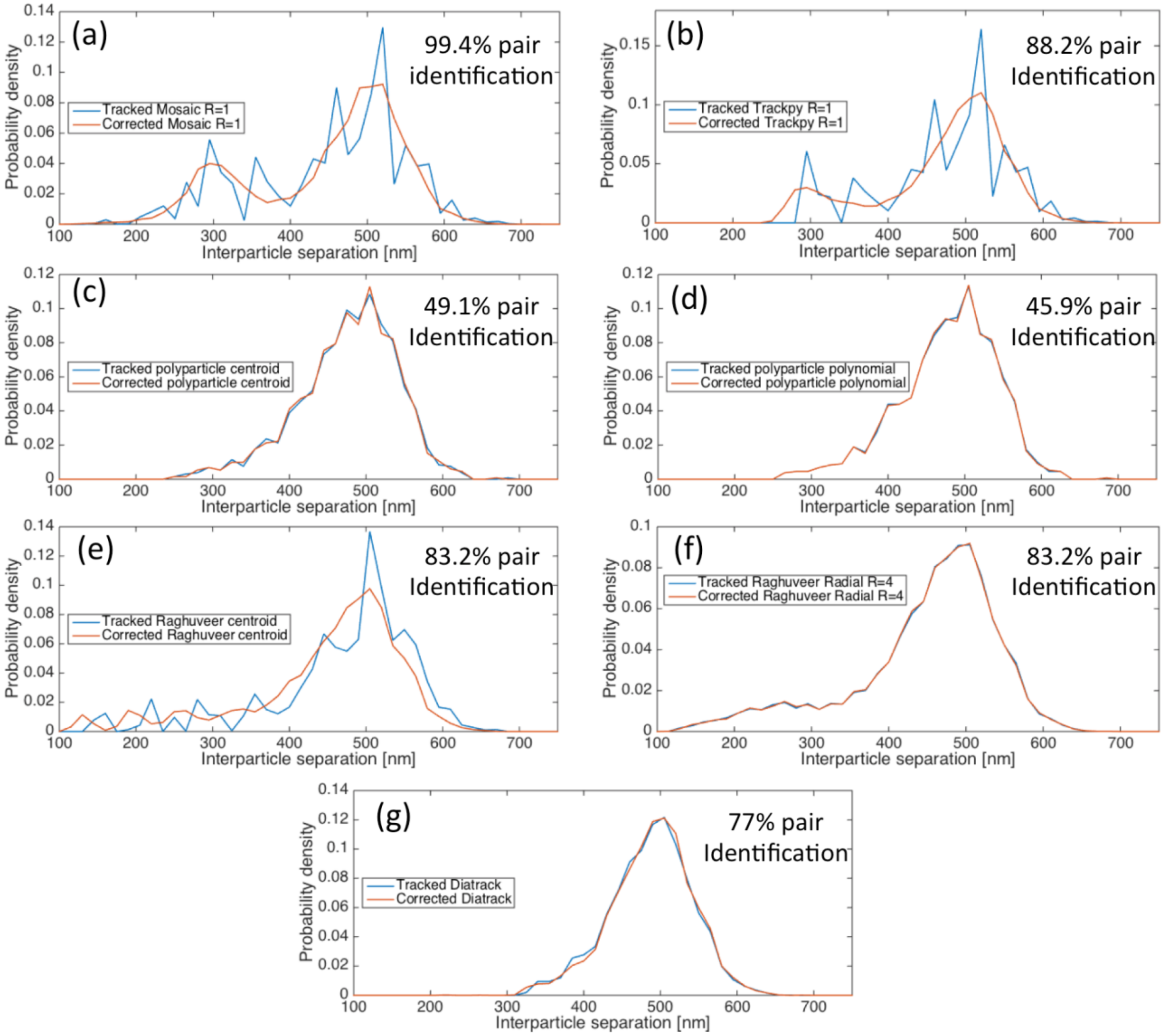


**Figure S8**. Probability distributions of interparticle separations of a pair of Ag NPs in a Gaussian trap using the same tracking algorithms as Figure S7. The distributions shown are for the following algorithms: (a) Mosaic algorithm for imageJ (b) Trackpy algorithm version 0.3.2 (c) Centroid output from the polypixel algorithm (d) polynomial output from the polypixel algorithm (e) Radial calculation from the Raghuveer software package (f) Centroid calculation from the Raghuveer software package (g) Diatrack 3.05 algorithm. Pair identification is the percentage of the frames in which two particles were identified.

We compared the output position lists obtained from the different tracking algorithms by calculating the Cartesian separation between the two particles across the entire video. Figure S8 shows the distribution of

interparticle separations before (blue curves) and after (red curves) SPIFF correction for each algorithm. Most of the algorithms were poor at identifying 2 particles when they were closer than roughly 5 pixels (350 nm). This is evident in comparing the dual-peaked distributions obtained in panels (a) and (b) to the single-peaked distributions in panels (c-g). The identifications for smaller separations are present at best as a long tail. This result is consistent with the identification percentages of the algorithms given above and in each panel in Figure S8. The Diatrack software suite for example (panel (g)), identifies two particles in 77% of the frames. The remaining 23% of the frames almost all are those in which the particles were too close to be separated and identified by the algorithm. As a result, the separation distribution consists of a single peak at the optical binding separation. This effect is also evident in the distribution of separations obtained by Trackpy in panel (c), which drops to zero at separations less than 300 nm. Since the minimal separation for this algorithm was defined as 4 pixels (*diameter + 1* pixel), it follows that the smallest observed separation would be $4\, pixels \times 72 \,{}^{nm}/_{pixel} = 288\, nm.$

A second point worth noting is the effect that the SPIFF correction has on the distributions. When the tracked data is biased and pixel locking is evident (panels (a), (b) and (e)) we observe that the separation distribution is spiky and discontinuous. The SPIFF correction acts to smooth the curves to a more physical distribution as discussed in the main text. This is similar to what was shown in the main text in Figures 2(c), 3(g) and 4(c). Furthermore, when the meta-pixel is uniform (panels (c), (d), (f) and (g)) the SPIFF correction introduces minor changes to the interparticle separation distribution, as expected.

The work in this section is not an exhaustive comparison of the behavior of particle tracking software and the effect of SPIFF correction. Nonetheless, it does demonstrate that SPIFF correction is a useful tool that can be applied to the localization data obtained from any tracking algorithm. The particle tracking problem we analyze demonstrates a trade-off between a pixel locking error, which can be corrected by the SPIFF algorithm, or an unkown and uncorrectable inaccuracy of missed interparticle separations!

**5. Improving the accuracy of interparticle distance determination beyond global SPIFF correction:**

Close examination of the distribution of tracked particle positions shown in Figure 2(c) in the main text and again in Fig. S9(a) reveals that when the interparticle separation becomes small, and the intensities from the two particles overlap on the detector, the identification percentage, that is the percentage of frames in which two particles are identified decreases, and the meta-pixel distributions are not evenly biased around the pixel center. The skew in the tracked localizations is demonstrated in Figure S9, which shows a single row in the distribution of localizations from the main text. The resulting SPIFF-corrected particle positions are skewed towards the edge of the pixel in the frames in which the interparticle separation is small instead of being evenly distributed throughout the pixel. This skewing bias cannot be

corrected with a single “one size fits all” SPIFF inverse function. Thus, an error remains in the SPIFF corrected data.

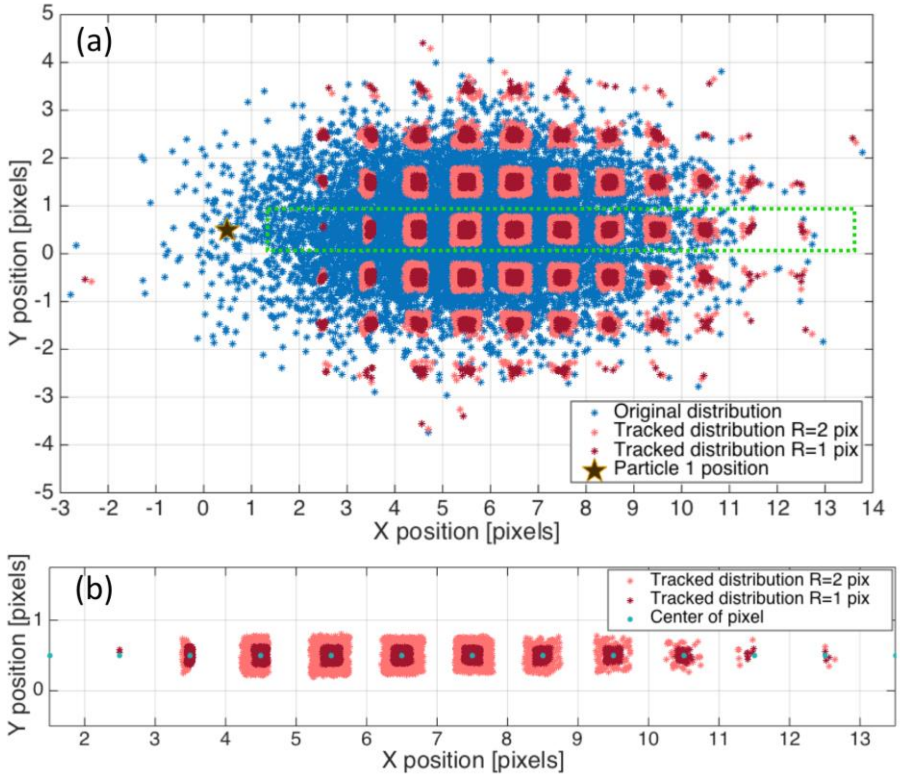


**Figure S9.** (a) Distribution of localizations for particle 2 (blue dots) in relation to the fixed position of for particle 1 (marked as black star). These distributions are the basis for analysis of pixel locking errors and SPIFF correction, as described in main text and shown in Figure 2(c). Red and pink dots show the distribution of tracked particles using the Mosaic nonlinear least-squares Gaussian fitting algorithm with a window of $R=1$ and $R=2$, respectively. Only frames where both particles are identified are considered. Particle 1 was localized at (0.5,0.5) of the pixel coordinate system due to the Mosaic algorithm. The dotted green rectangle outlines a selected range highlighted in the panel (b). (b) A single row from the distribution map shown in (a) and the main text in Fig. 2(c). All localizations that were found between y = 0 and y = 1 pixels. Cyan dots designate the pixel centers. Some of the distributions localized to the pixels exhibit a skew. In this figure 1 pixel is equal to 72x72 nm in size.

Figure S9 shows that the distribution of localizations becomes skewed around the pixel center as the interparticle separation decreases. The overlap in the intensities of the two particles skews the meta-pixel distribution to a different separation. This is particularly evident for particles that are localized to pixels x=2 and x=3, which are very close to the position of particle 1.

This non-uniform biasing error can easily be corrected in this case where one particle is fixed by applying the SPIFF algorithm on a local, pixel-specific meta-pixel distribution, rather than on the global distribution taken from the entire video. In doing so one needs to evaluate Eqn. (1) in the main text relative to the center of mass of the metal-pixel distribution for that specific pixel rather than relative to

the center of mass of the meta-pixel distribution from the entire tracked video or relative to the pixel center. However, this solution only works when one of the objects in the image is fixed throughout the video and, therefore, the calculated position of the second particle uniquely determines the interparticle separation. However, in most experimental tracking scenarios all particles are free to move and a given interparticle separation does not correspond to a specific pixel position. Thus, the meta-pixel distribution of any given pixel will be composed of a large set of data-points originating from many different interparticle separations. As a result, the local meta-pixels will not be skewed and pixel-specific bias and correction information is not available.

Instead, further improvement to the localization accuracy beyond application of the SPIFF correction algorithm can be achieved by determining the separation (distance)-dependent error. This can be done by fitting the error data (i.e. the difference between the tracked particle separations and the corresponding true separation values) obtained from the analyzed synthetic frames and applying it to measurements that are performed under the same experimental conditions as the synthetic data are calculated for. As an example of this we use the simulated particle trajectories described in the main text and in Figure 3(h), calculate the mean error in separations as a function of the true interparticle separation, and fit it to a cubic function as shown in Fig S10.

Figure S10 shows the difference between the true interparticle separation, $d_{true}$, and the tracked interparticle separation, $d_{estimated}$, sorted by interparticle separation (red connected dots). We also plot this for the SPIFF-corrected separations (black dots) and fit their distribution to a cubic function that is overlayed on the distributions (blue curve). $d_{true}$ - $d_{estimated}$ is also known as the distance error. As can be seen from the black dots, the error is largest when the interparticle separation is ~5 pixels and reduces to 0 when the particles are separated by more than 7 pixels. Therefore, the cubic fit shown in the figure (blue) provides information for further correction of the interparticle separations obtained from tracked experimental datasets where there is no ground-truth data to compare to.

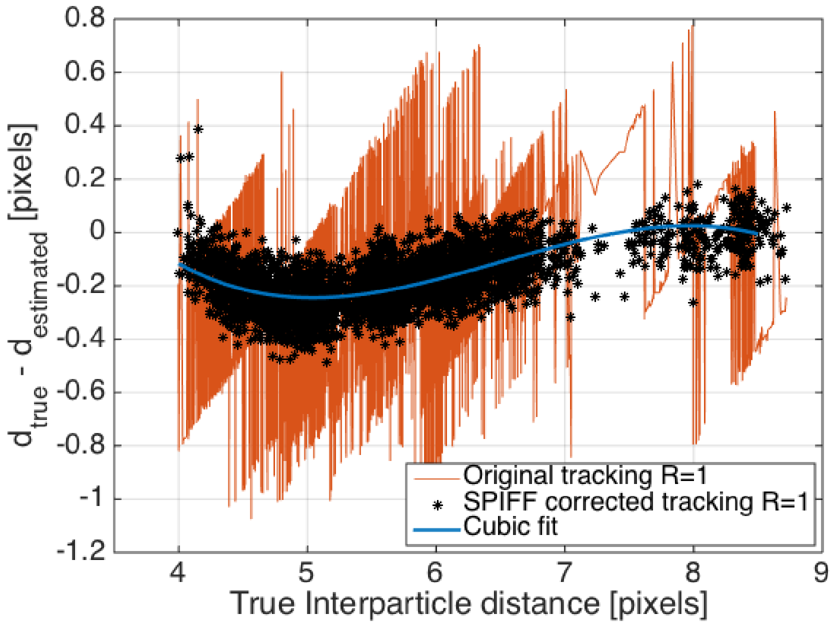


**Fig S10**. Error in interparticle separation defined as $d_{true}$ - $d_{estimated}$ for the tracked (red) and SPIFF corrected (black) synthetic data, as well as a cubic fit to the SPIFF corrected data (blue curve). See text for details.

The cubic fit (blue) provides additional information about how the imaging system skews the particle position as a function of the interparticle separation and can be used to further improve the accuracy of localization in experimental data. Since the synthetic images, and all observations obtained from them are based on the experimental results, we can now use the cubic fit shown in Fig. S10 to correct (or fit) data from (ED-LD) simulation and experiment. We calculate the fitted separation $d_{fitted}$ for a given frame $n$, as

$$d_{fitted}(n) = d_{estimated}(n) + g(d_{estimated}(n)), \qquad (1)$$

where $g(d)$ is the cubic fit of the distance error as a function of interparticle separation. This fit that has been extracted from the data and is shown in Figure S10. The results of applying the fit to the data are shown in Fig S11, which presents the time evolution of the separation as well as its distribution before and after the fitting. Where applicable, the corrected data are compared to the original data. The improvement is significant. Note that when the interparticle separation is relatively large (as seen in the left side of Fig. S11(a), and the green curve in Figure S11(c)) the cubic fit is small and there is a negligible difference between the SPIFF corrected and the error- fitted interparticle separation values. This is also evident in the probability distributions seen in Fig S11(b,d), which show that the cubic fit has a strong effect on the small interparticle separations (left side of the panels S11(b,d)), and a much weaker effect on the large interparticle separations (right side of panels S11(b,d)). It is important to note that the experimental data analyzed in panels S11(c,d) cannot be compared to ground truth data, as the “true”

particle positions are unknown (as opposed to the simulated data in panels S11(a,b)). Therefore, the accuracy of the correction for experimental data warrants further investigation.

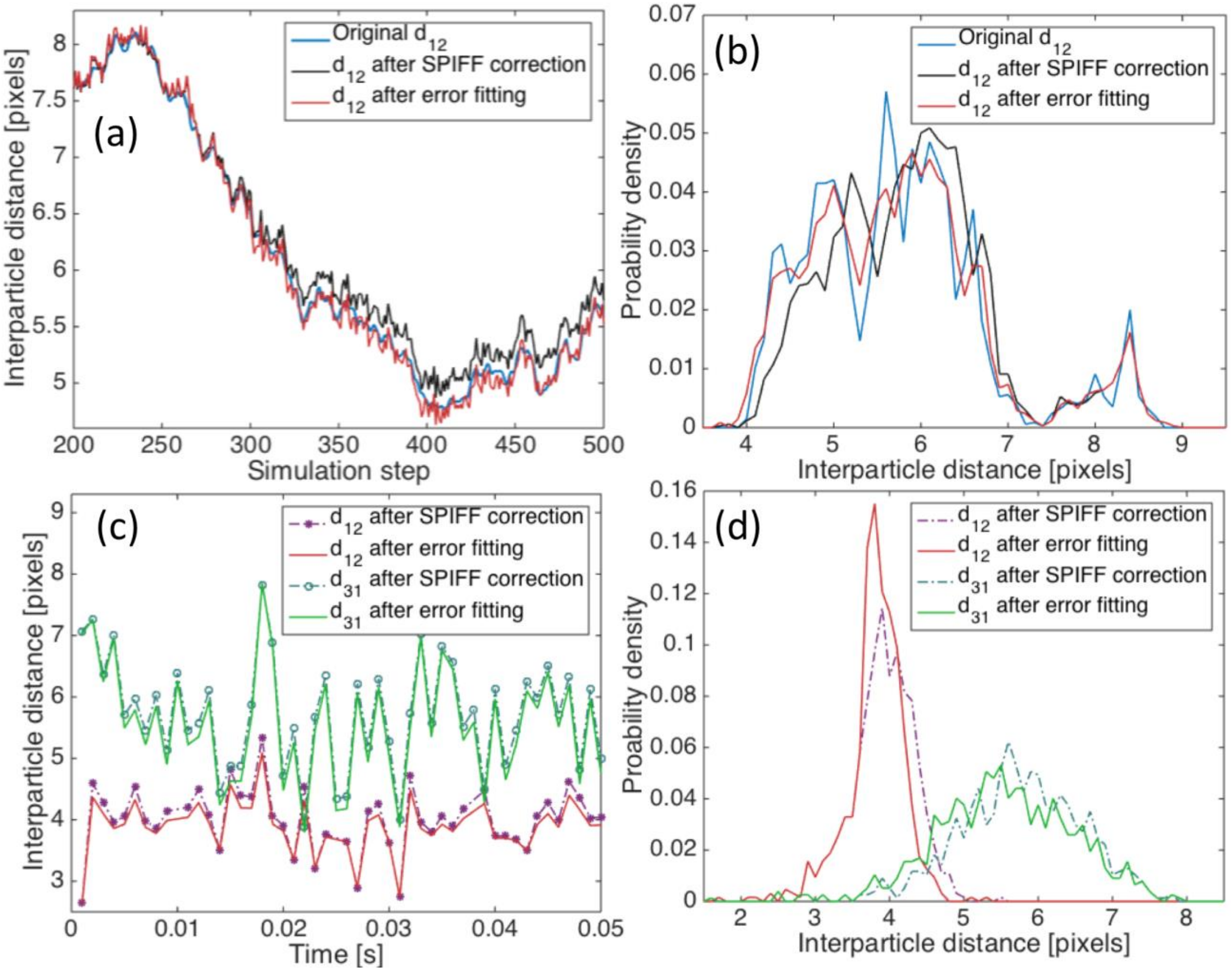


**Figure S11.** Further improvement of experimental and simulated interparticle separation measurement by application of cubic error fitted data as shown in Fig. S4. (a) Time dependent interparticle separation taken from the ED-LD simulation set described in the main text and in Figure 3. Panel (a) shows the interparticle separation calculated directly after SPIFF correction (black) and after further improvement using fitted error data (red) and how both compare to the original data (blue). Note how the fitted curve (red) improves the fidelity of the measured separation to the true data compared to the SPIFF corrected curve (black), especially for smaller interparticle separations. (b) Probability distribution of interparticle separations for the original data compared to the SPIFF corrected and fitted data. Experimental data analysis – time dependent separation (c) and probability distribution (d) for data taken from the experimental video described in Figure 4 of the main text. The definitions of the separation $d_{12}$ and $d_{31}$ are given in the main text and in Figure 4.

The results of this section are a simple demonstration of a new approach that provides additional improvement of the accuracy of particle tracking data. The analysis presented in this section demonstrates the potential for using the information gained from simulated data to further improve experimental

results. Although the results presented here are based on our analysis of imaging of 150 nm diameter Ag particles in the optical setup described in Section 1, the approach is general and can be applied to any imaging system and tracked object. Thus, we believe that using synthesized data, along with more sophisticated fitting methods, will improve the localization accuracy of experimental data, and warrants further research.

**6. Preparation of trajectories and synthetic images of single fluorescent protein tracking:**

Lin et al [9] describe tracking single fluorescent proteins moving on a single ds-DNA strand under different flow conditions to extricate a mechanism of motion. They used the tracked data to understand and model the protein behavior by fitting their data to a drift-diffusion Langevin equation and solving it to obtain a probability density function (PDF) that describes the distribution of step sizes for a flow-biased walk:

$$P(x,t) = \frac{1}{\sqrt{4\pi Dt}} exp - \left[\frac{1}{4Dt}\left(x - \frac{\alpha ut}{\gamma}\right)^2\right], \qquad (2)$$

where, $P$ is the probability density of step sizes, x, and as a function of the time step, $t$, with flow rate $u$, drag coefficients $\alpha$ and $\gamma$ and $D = kT/\gamma$ is the diffusion coefficient of the protein on DNA.

Using this equation and parameter values from ref. 9, we generated lists of $10^4$ displacement values for the experimental flow values reported in the paper (2,3 and 4 µl/min) for a time step of 0.5ms. We then generated simulated trajectories of the protein under different flow conditions by cumulatively summing these steps (see Figure S12). This is essentially Monte-Carlo sampling of a Wiener process. By using a time step of 0.5ms, we can effectively sample the motion of the particle at 2000 FPS, a value that is two orders of magnitude faster (larger) than what was reported in the CCD-based experiments (note that this frame rate can be achieved with a sCMOS camera in a small region of interest by cropping to reduce the portion of the array being used).

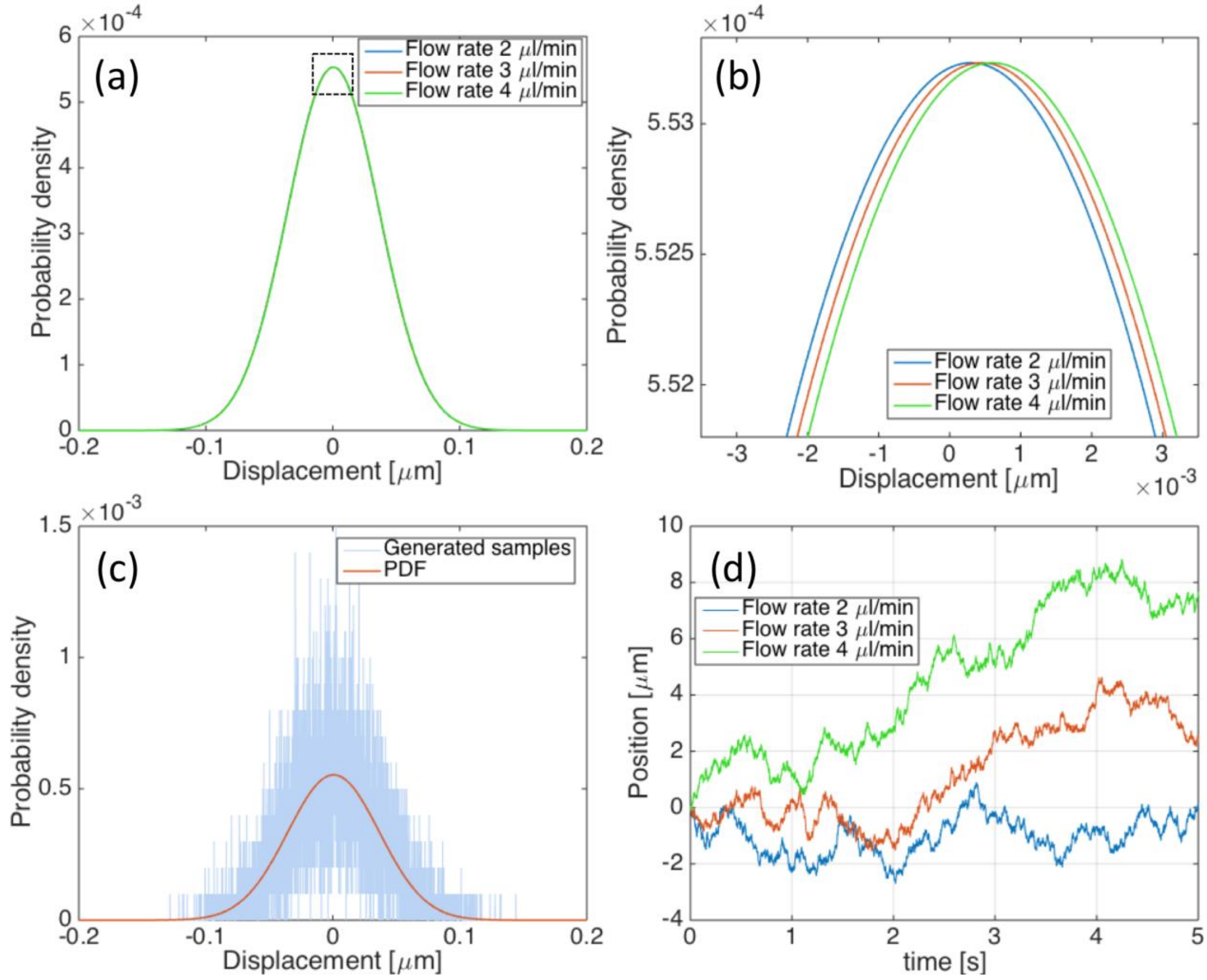


**Figure S12**. (a) Probability density function (PDF) of step sizes for a time step of 0.5ms and three different flow rates (2, 3 and 4 ul/min) used in the original experiment by Lin et. al [3]. (b) Zoomed in view of panel (a) of the region marked by the black rectangle. Note the slight difference in the positions of the maxima for different flow rates. (c) PDF for a flow of 4 ul/min (red line) along with a normalized histogram of $10^4$ random samples from the distribution (blue bars). The random samples were summed to create trajectories. (d) Particle trajectories for flow rates of 2, 3, and 4 ul/min (blue, red and green respectively). The trajectory for 4 ul/min was used to synthesize the images for particle tracking.

These trajectories were then used to create a list of coordinates that was input into the image synthesis procedure described above. Similar to what was described in section 2 of the SI, we analyzed the experimental frames and extracted the mean intensity and standard deviation of the background pixels. These exhibited a Gaussian intensity distribution in addition to a few counts of high intensity pixels that are due to other fluorescent proteins streaking though the frame outside the plane of focus. The background of the synthetic frames was based on the Gaussian part of this distribution. We also analyzed the mean value and variance of the intensity of the particle. In addition, we fitted a 2D Gaussian function

to images of the particle intensities (in pixels on the detector) to extract the mean point spread function of the experimental imaging system. We then used these extracted values to simulate the particle intensity at the desired location on the frame.

However, for most experimental frames (images) the protein intensity distribution is not Gaussian due to blinking and fluctuations of the single molecule intensity and presumably some fast motion (see Fig S13(a)). To simulate this effect, we added an additional intensity filter that, for the pixels around the protein location, either increased or attenuated the pixel intensity by a random, normally distributed, value. This value determines the strength of the filter and acts to create a less uniform intensity map around the particle position. Examples of a frame with no additional filtering and with mild (variance of 10%) strong (variance of 25%) and extreme (variance of 50%) filtering values are shown in Figure S13. The analysis described in the main text was performed on the strongly filtered frames, except for Figure 5(e). In that panel, which shows the original and SPIFF-corrected trajectories, we chose the values of the mildly filtered frames to improve the clarity of the trajectory shown in figure 5(e).

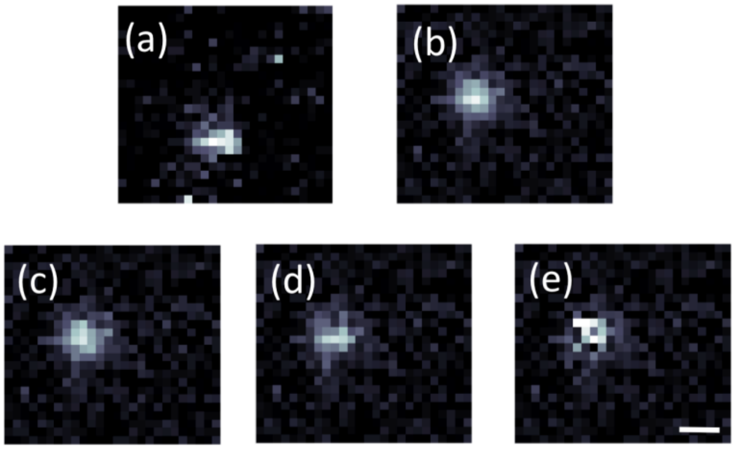


**Figure S13**. Representative images of fluorescent single molecule and synthetic images. (a) Experimental image. (b) Synthesized image with no additional filtering. (c) Synthesized image with a mild filtering (variance of 10% in the intensity of pixels associated with the particle). (d) Synthesized image with a strong filtering (variance of 25% in the intensity of pixels associated with the particle). (e) Synthesized image with extreme filtering (variance of 50% in the intensity of pixels associated with the particle). Scale bar represents 400 nm.

Application of these filters acts to replicate the noise in the experimental images. We observe pixel locking in all cases. The application of the SPIFF correction algorithm improves the fidelity of the MSD to the true values even when there is no filter applied (see Figure S14). However, Figure S14 shows that as the noise from the filtering process increases, the fidelity of the tracked trajectory to the true value decreases. Despite this, the SPIFF correction still reduces the error in the MSD by up to 0.25 pixels, demonstrating that SPIFF correction is effective even when applied to very noisy experimental image data.

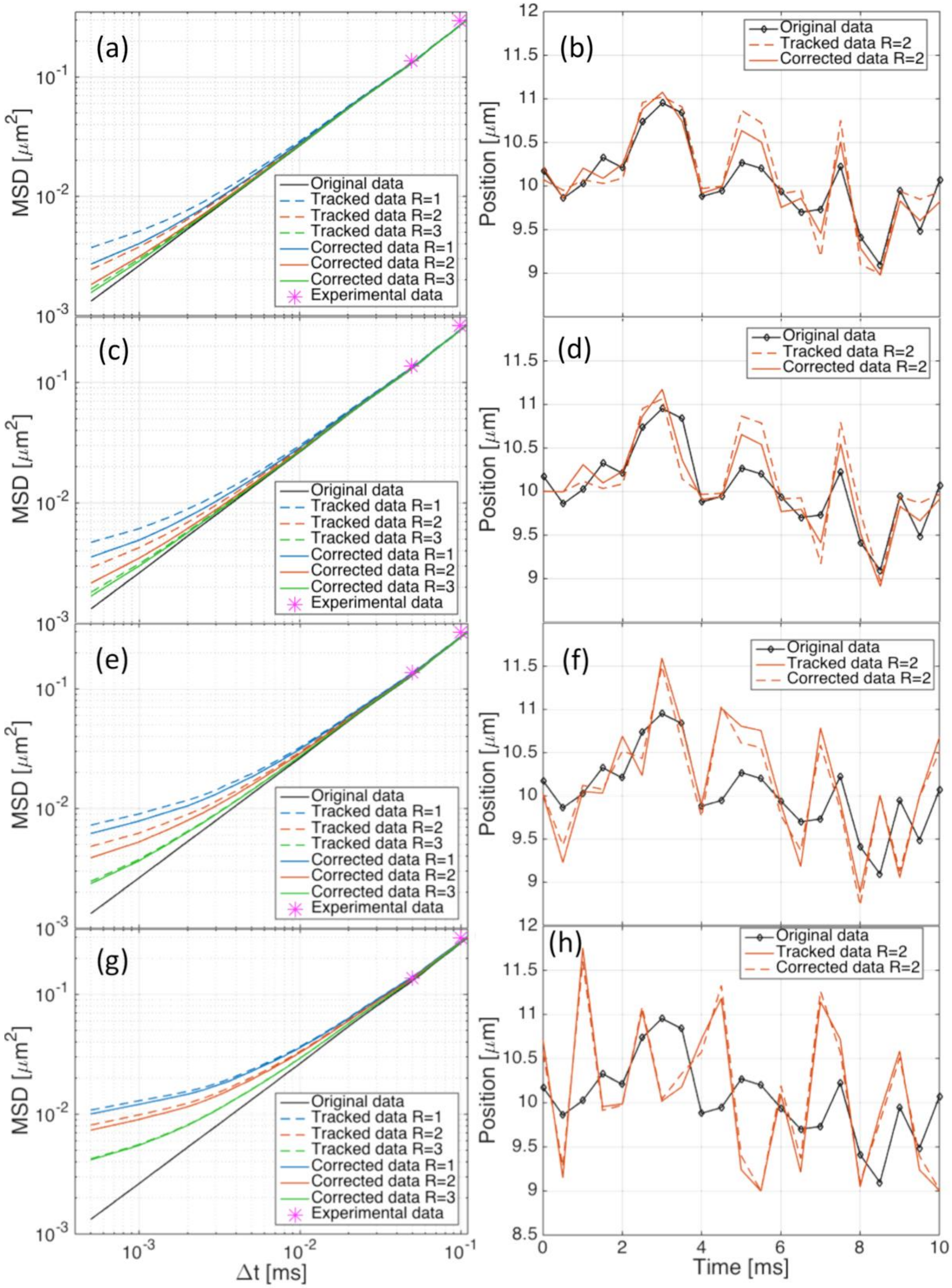


**Figure S14.** Mean Square Displacement (MSD - left column) and trajectory (right column) plots obtained from tracking images that were synthesized under different filtering conditions. (a,b) No filtering (c,d) Mild filtering (e,f) Strong filtering (g,h) extreme filtering.

**7. The effect of decreased frame rate on fluorescent molecule trapping:**

As was mentioned in the main text, when we applied the SPIFF algorithm to the original experimental single fluorescent protein data, which was captured at 20FPS, we did not observe a consistent improvement in the MSD; i.e. the difference between the MSD calculated from the original and the corrected values varied between negative and positive values. However, when we analyzed this for the synthesized 2000FPS data, the SPIFF corrected MSD values were consistently closer to the true values than those calculated from the pixel-locked tracked data. An example of the fidelity of the high frame rate data is shown in Figure 5(f), which shows that the difference between the original MSD and the corrected MSD is positive for all time separations, in contrast with Figure 5(c), which shows the experimental data and has both positive and negative values. The reason for the pronounced improvement of the SPIFF correction for high frame rate videos relative to what was seen in the experimental data is that the original data was sampled at 20FPS, and as a result, the particle displacement between frames is larger than the pixel locking error. Therefore, applying the SPIFF algorithm could either increase or decrease the MSD. If the data were sampled at a high enough frame rate, the mean particle displacement between frames will be sub-pixel and the additional sub-pixel correction obtained through the SPIFF algorithm is more likely to improve the accuracy of the MSD.

In order to verify this conjecture, we selected two subsets from the synthetic tracking data: in one subset we selected every $100^{th}$ frame and in the other we selected every $10^{th}$ frame, thus reducing the 2000FPS data to 20FPS and 200FPS respectively. The results of the MSDs calculated from these data subsets are shown in Figure S15. As expected, decreasing the sampling rate causes the mean displacement between frames to exceed 1 pixel per frame, thereby reducing the significance of the SPIFF correction. Note that for the data sampled at high frequency (Fig. S15c) the difference is always positive, which means that the SPIFF algorithm corrects the MSD value by a significant amount relative to the particle motion from frame to frame. On the other hand, for the data at lower sampling frequencies, the difference can be either positive or negative, similar to what was shown in the main text. This means that the particle motion is >1 pixel, causing the SPIFF correction to be insignificant. We referred to this latter situation as *temporarily undersampled*. As stated in the Conclusions, the error in accuracy can be associated with biases in tracking, but we now see that temporal undersampling results in an error that is not corrected by the SPIFF algorithm. This point requires further investigation.

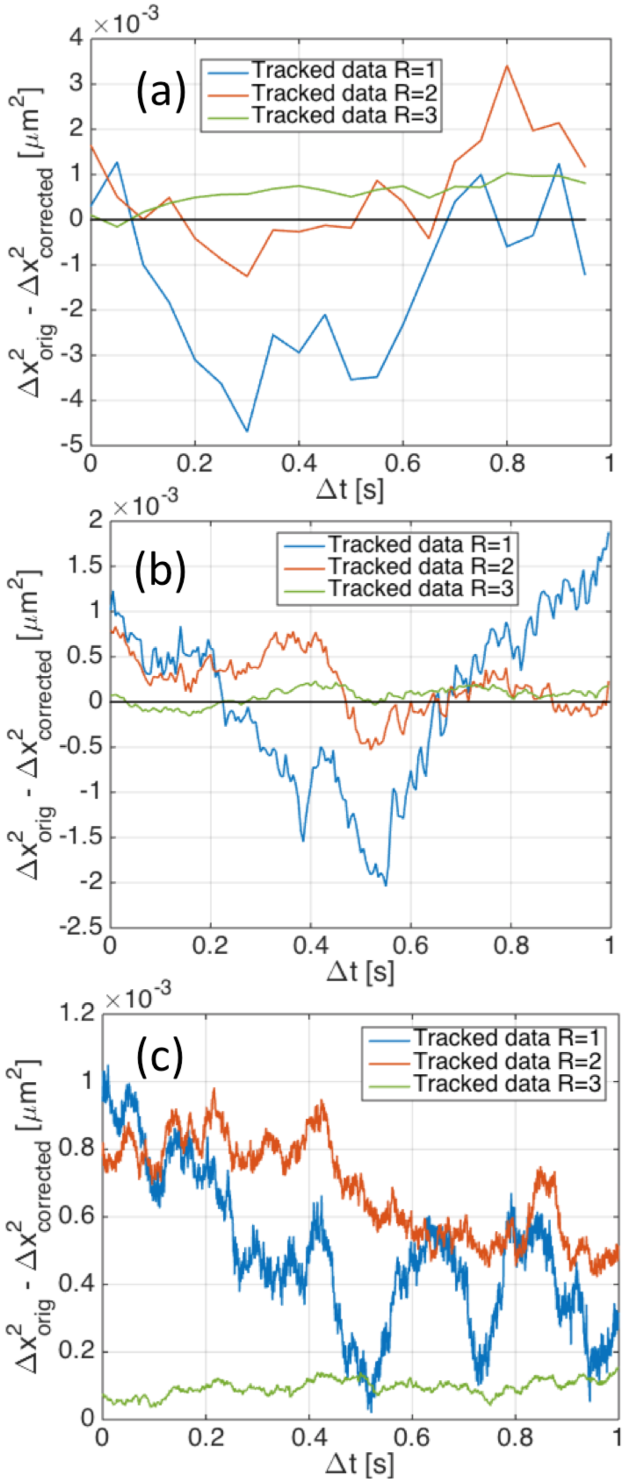


**Figure S15.** Difference between the original and the SPIFF-corrected MSD values for synthesized trajectories analyzed at (a) 20FPS (b) 200 FPS (c) 2000 FPS. Note that for the high frequency sampled data the difference is always positive, implying that the SPIFF algorithm corrects the error and brings the estimated MSD closer to the behavior expected for a Brownian random walk.

**8. Matlab implementation of the SPIFF algorithm:**

```
function corrected_p = SPIFF_sort_published(p_in,SPIFF_x,SPIFF_y)

%A function that returns a SPIFF-corrected particle list based on the
%position list given in p_in, and (potentially) the x and y components
%of the SPIFF-distribution (SPIFF_xx and SPIFF_y, respectively)
%Assumption: the input p_in is a vector of size 2xn size with the
%x-coordinates in the 1st column and the y coordinates in the 2nd column.

%%%%%%%%%Generate SPIFF distribution (if necessary):%%%%%%%%
if nargin == 0  %If the function was called empty
    corrected_p = [-1,-1];
    return
elseif nargin ==1   %No metapixel data was given
    SPIFF_x = p_in(:,1)-floor(p_in(:,1));
    SPIFF_y = p_in(:,2)-floor(p_in(:,2));
end

%%%%%%%%%%%%SPIFF correction %%%%%%%%%%%%%%%%%%
%Now calculate the positive and negative probability density distributions
%(P(x_e) in the manuscript - eq. (1), and in the original manuscript
%by Burov et. al. Eq. (6)). Calculation is relative to the pixel center (0.5,0.5);
SPIFF_x_plus = SPIFF_x(SPIFF_x>=0.5);
SPIFF_y_plus = SPIFF_y(SPIFF_y>=0.5);
SPIFF_x_minus = SPIFF_x(SPIFF_x<0.5);
SPIFF_y_minus = SPIFF_y(SPIFF_y<0.5);

p_x_plus = sort(SPIFF_x_plus);
p_x_minus = sort(SPIFF_x_minus);
p_y_plus = sort(SPIFF_y_plus);
p_y_minus = sort(SPIFF_y_minus);

%The SPIFF corrected positions go here:
corrected_p = zeros(size(p_in));

%Seperates the positive and negative parts of the SPIFF in both x and y.
% for each one of the biased points, calculates the amount of points
% below its value.

for ww = 1:length(p_in)
    %Treat position list for particle 1
    if p_in(ww,1) == 0
        continue
    end
    x1_curr = p_in(ww,1);
    y1_curr = p_in(ww,2);
    SPIFF_x_curr = x1_curr - floor(x1_curr);
    SPIFF_y_curr = y1_curr - floor(y1_curr);
    if SPIFF_x_curr>=0.5
        %a. Get percentile value:
        tmp_x = sum(p_x_plus<=SPIFF_x_curr)/length(p_x_plus);
        %b. Bring back to required position:
        new_SPIFF_x = tmp_x/2;
        %if the position is positive (above the floor) it stays.
        corrected_p(ww,1) = ceil(x1_curr) + new_SPIFF_x;
```

```
    else
        %a. Get percentile value (how many particles are closer to center):
        tmp_x = sum((p_x_minus)>=(SPIFF_x_curr))/length(p_x_minus);
        %b. Bring back to required position:
        new_SPIFF_x = tmp_x/2;
        %If the position is negative (below the floor) it is moved by 1.
        corrected_p(ww,1) = ceil(x1_curr) - new_SPIFF_x;
    end

    if SPIFF_y_curr>=0.5
        %a. Get percentile value:
        tmp_y = sum(p_y_plus<=SPIFF_y_curr)/length(p_y_plus);
        %b. Bring back to required position:
        new_SPIFF_y = tmp_y/2;
            %if the position is positive (above the floor) it stays.
        corrected_p(ww,2) = ceil(y1_curr) + new_SPIFF_y;
    else
        %a. Get percentile value (how many particles are closer to center):
        tmp_y = sum((p_y_minus)>=(SPIFF_y_curr))/length(p_y_minus);
        %b. Bring back to required position:
        new_SPIFF_y = tmp_y/2;
        %If the position is negative (below the floor) it is moved by 1.
        corrected_p(ww,2) = ceil(y1_curr) - new_SPIFF_y;
    end
end

%%%%%%%%%%%%%%%%%%%%%%%%%%%%%%%%%%%%%%%%%%%%%%%%%%%%%%%%%%%%%%%%%%%%%%%
```

**7. List of videos:**

**Video S1** – Video of synthetic images (frames) of two Ag particles trapped in a linearly polarized Gaussian trap. Trajectories were obtained using ED-LD simulation method referenced in text, and used to synthesize movie.

**Video S2** – Experimental video of three particles in linearly polarized Gaussian trap.

**Video S3** – Representative Experimental video of fluorescent single molecule trajectory.

**Video S4** –Videos of a trajectory of synthetic images of particle motion with different filtering levels for comparative purposes - no filtering, mild filtering, strong filtering and extreme filtering (labels in video).

**Reference list:**


1. Figliozzi, P. *et al.* Driven optical matter: Dynamics of electrodynamically coupled nanoparticles in an optical ring vortex. *Phys. Rev. E* **95,** 22604 (2017).
2. Pawley, J. B. in *Handbook of biological confocal microscopy* 59–79 (Springer, 2006).
3. Yan, Z., Sajjan, M. & Scherer, N. F. Fabrication of a Material Assembly of Silver Nanoparticles Using the Phase Gradients of Optical Tweezers. *Phys. Rev. Lett.* **114,** (2015).
4. Sbalzarini, I. F. & Koumoutsakos, P. Feature point tracking and trajectory analysis for video imaging in cell biology. *J. Struct. Biol.* **151,** 182–195 (2005).

5. Allan, D., Caswell, T., Keim, N., & van der Wel, C. (2016, August 19). trackpy: Trackpy v0.3.2. Zenodo. http://doi.org/10.5281/zenodo.60550

6. Rogers, S. S., Waigh, T. A., Zhao, X. & Lu, J. R. Precise particle tracking against a complicated background: polynomial fitting with Gaussian weight. *Phys. Biol.* **4,** 220–227 (2007).

7. Parthasarathy, R. Rapid, accurate particle tracking by calculation of radial symmetry centers. *Nat. Methods* **9,** 724–726 (2012).

8. Vallotton, P. & Olivier, S. Tri-track: Free Software for Large-Scale Particle Tracking. *Microsc. Microanal.* **19,** 451–460 (2013).

9. Lin, Y. *et al.* Using the bias from flow to elucidate single DNA repair protein sliding and interactions with DNA. *Biophys. J.* **96,** 1911–1917 (2009).
